# Using invariom modelling to distinguish correct and incorrect central atoms in ‘duplicate structures’ with neighbouring 3*d* elements

**DOI:** 10.1107/S2052520617010745

**Published:** 2017-09-29

**Authors:** Claudia M. Wandtke, Matthias Weil, Jim Simpson, Birger Dittrich

**Affiliations:** a Institut für Anorganische Chemie der Universität Göttingen, Tammannstrasse 4, Göttingen D-37077, Germany; b Technische Universität Wien, Getreidemarkt 9/164-SC Stg 1, A-1060 Wien, Austria; c University of Otago, PO Box 56, Dunedin, New Zealand; d Heinrich-Heine Universität Düsseldorf, Institut für Anorganische Chemie und Strukturchemie, Material- und Strukturforschung, Gebäude: 26.42, Universitätsstrasse 1, 40225 Düsseldorf, Germany

**Keywords:** invariom modelling, duplicate structures, metal atom identification

## Abstract

Modelling coordination compounds has been shown to be feasible using the invariom method; for the best fit to a given set of diffraction data, additional steps other than using lookup tables of scattering factors need to be carried out. Here such procedures are applied to a number of ‘duplicate structures’, where structures of two or more supposedly different coordination complexes with identical ligand environments, but with different 3*d* metal ions, were published. However, only one metal atom can be plausibly correct in these structures, and other spectroscopic data are unavailable. Using aspherical scattering factors, a structure can be identified as correct from the deposited Bragg intensities alone and modelling only the ligand environment often suffices to make this distinction. This is not possible in classical refinements using the independent atom model. Quantum-chemical computations of the better model obtained after aspherical-atom refinement further confirm the assignment of the element in the respective figures of merit.

## Abstract 2

Aspherical atom refinement with conventional data sets is now possible for coordination compounds. As example structures, a number of pairs of published structures, where the element-type assignment of the metal was unclear, have been re-investigated. Identification of which structure is correct can be made from the deposited Bragg intensities alone.

## 1. Introduction   

Structure determination from single-crystal X-ray diffraction (XRD) has become a mature technique in recent decades (Spek, 2009 ▸). Thus, the number of crystal structures published each year has increased exponentially, as shown by the statistics of the Cambridge Structural Database (CSD; Groom *et al.*, 2016 ▸), where most published structures are deposited. Very successful validation procedures concerning crystallographic information exist in the form of the automated *checkCIF* procedure (http://checkcif.iucr.org/), which relies on the program *PLATON* (Spek, 2003 ▸, 2009 ▸). However, assessing the chemical/physical correctness of a crystal structure remains challenging and ultimately requires human judgement. While missing or misplaced H atoms and incorrectly assigned atom types, in general, can often be identified already by specific indicators deduced from a structural model by *checkCIF*, problematic atom-type assignments for metal atoms are not easily recognized. Even possible fraud can sometimes be detected from an analysis and comparison of reflection data (Harrison *et al.*, 2010 ▸; Zhong *et al.*, 2010 ▸; Liu *et al.*, 2010 ▸; IUCr Editorial Office, 2011*a* ▸,*b* ▸, 2012*a* ▸,*b* ▸)^1^. Other useful tools for structure validation rely on deposited structures and a statistical analysis of bonding. Here, deviations from known ranges of bond lengths that exceed a statistically significant threshold can be identified, *e.g.* with the program *Mogul* (Bruno *et al.*, 2004 ▸, 2011 ▸). A more general discussion of structure validation (from the viewpoint of macromolecular structure determination) has been given by Dauter *et al.* (2014) ▸.

What automated validation procedures cannot currently reliably provide is an answer to the question of chemical correctness. Especially when several crystallographically plausible structural models fit diffraction data equally well is there a need for the correct chemical interpretation (Haaland, 1994 ▸). Coordination compounds of *d*-block elements with their rather high flexibility concerning ligand arrangement often provide an interesting challenge: the situation can arise that the wrong element leads to better agreement statistics in least-squares refinements using the independent atom model (IAM) (Dittrich *et al.*, 2015 ▸). The IAM can thus fail to distinguish between neighbouring elements in such com­pounds and may lead to the wrong assignment, especially when single-crystal XRD is the only analytical technique being relied upon. Therefore, a method improvement indicating the correct choice of metal among neighbouring elements would increase the value of single-crystal XRD as an analytical tool.

Aspherical scattering factors have proven their value in charge-density research (Spackman & Brown, 1994 ▸; Tsirelson & Ozerov, 1996 ▸; Coppens, 1997 ▸; Spackman, 1998 ▸; Koritsánszky & Coppens, 2001 ▸). Scattering-factor databases^2^ provide the technical functionality required to replace the IAM for conventional data (Dittrich *et al.*, 2006*b* ▸, 2009 ▸; Bendeif & Jelsch, 2007 ▸; Sanjuan-Szklarz *et al.*, 2016 ▸). However, a broad user base is still lacking, and this is probably also due to the need to obtain, learn and use expert programs to describe the aspherical electron-density distribution ρ(**r**) (EDD) that is not taken into account in the IAM. Scattering factor databases rely on implementations of the Hansen–Coppens multipole model^3^ (Hansen & Coppens, 1978 ▸) and require local-atomic coordinate systems, adding complexity to the process of least-squares refinement. An alternative approach uses extremely localized molecular orbitals (ELMO’s) (Meyer *et al.*, 2016 ▸) and it will be interesting to see how this will develop.

Using aspherical scattering factors can provide additional value in answering particular research questions. They permit the provision of additional properties from molecular EDD directly from crystal structure determinations (*e.g.* Holstein *et al.*, 2012 ▸). Their use also results in more accurate and precise structures compared to the IAM, as advocated early on (Brock *et al.*, 1991 ▸). Such improvements manifest themselves in deconvoluted atomic displacement parameters (ADPs) (Jelsch *et al.*, 1998 ▸; Dittrich *et al.*, 2008 ▸) and reduced differences of mean-square displacement amplitude (DMSDA) values in the bond direction (Hirshfeld, 1976 ▸; Rosenfield *et al.*, 1978 ▸; Dittrich *et al.*, 2005 ▸). Coordinate shifts due to asphericity (Coppens *et al.*, 1969 ▸; Volkov *et al.*, 2007 ▸; Dittrich *et al.*, 2007 ▸) are also corrected. Model improvements also result in lower standard uncertainties of all refined parameters, including the Flack (1983 ▸) parameter (Dittrich *et al.*, 2006*c* ▸), and better figures of merit, as shown in numerous articles, *e.g.* Bąk *et al.* (2011 ▸). While, in particular, the latter results are relevant to a general audience, research in this area may still be considered work in progress; apart from the above-mentioned issues of program availability and usability, it was unsatisfactory that most earlier efforts were directed towards crystals of molecular compounds consisting of light elements only.

With the increased accuracy from using aspherical scattering factors established, we can now tackle another weakness of conventional crystal structure refinement relying on the IAM, namely distinguishing between neighbouring elements in the periodic table. Eleven cases of ‘duplicate structures’ containing 3*d* metal atoms have been studied in this respect^4^. By ‘duplicate structures’ we mean pairs of structures reported to have different metal atoms, which appear on closer inspection to be duplicates of the same structure. These structures consist of coordination compounds that were published in the journal *Acta Crystallographica Section E*. They have identical unit-cell parameters [within realistic accuracy (Herbstein, 2000 ▸) and associated standard deviations], but different neighbouring 3*d* metal ions. Fortunately, this journal has required the deposition of diffraction data since its inception in 2001, and we investigate whether a distinction between correct and false structures is now possible in retrospect. These structures will be discussed in detail in *Results and discussion* (§3), and in the supporting information.

## 2. Methods and procedures   

### 2.1. Theoretical computations and new model compounds   

All model compounds in the generalized invariom database (Dittrich *et al.*, 2013 ▸) were re-optimized using the Minnesota DFT (density functional theory) functional M06 (Zhao & Truhlar, 2008 ▸) and the def2TZVP all-electron basis set (Weigend & Ahlrichs, 2005 ▸). This method/basis set combination can cover all elements up to bromine (krypton) and will be used throughout this article unless stated otherwise. Currently, all-electron basis sets are technically required to generate aspherical scattering factors (Dittrich *et al.*, 2005 ▸) using Fourier transform methods (Jayatilaka, 1994 ▸). In addition, the number of model compounds has been increased substantially to cover ligands common in coordination chemistry, as well as important aromatic model compounds that contain fluorine, chlorine and bromine^5^. As a result, the number of model compounds has now climbed close to 2000, giving over 4000 invariom scattering factors, a substantial increase compared to the number in the 2013 release of the generalized invariom database (Dittrich *et al.*, 2013 ▸). Although many model compounds are still missing, coverage has improved considerably for organic model compounds containing H, C, N, O, F, Cl and Br. Compiling data for structures of molecules containing S and P will require more work.

### 2.2. Special aspects of modelling coordination compounds   

Aspherical scattering factors transferred from the invariom database usually describe only the ligand environment, because the database mainly contains organic molecules. Directly bonded ligand atoms can usually be assigned manually to a coordination compound by ignoring the central metal atom^6^. In order to ultimately acquire the aspherical scattering factors for a complete molecule, *i.e.* including the central metal atom and the dative bonds formed by the directly bonded ligand atoms, a single-point DFT calculation is performed on the geometric model obtained after invariom refinement. The molecular EDD obtained this way is then projected onto the multipole model (‘whole-molecule’ scattering factors). For technical reasons, this projection is performed *via* ‘a detour through reciprocal space’: the molecular electron density is converted into structure factors by a Fourier transform process in a simulated diffraction experiment. This procedure is used both to generate entries in the invariom database and to tailor the scattering factors for the particular molecule being investigated. The following steps are then required (Dittrich *et al.*, 2015 ▸):

(i) the molecule to be computed by quantum chemistry is chosen, omitting any solvent and taking account of crystal symmetry for atoms on special positions;

(ii) placement of the molecule in an artificial unit cell;

(iii) Fourier transform into (simulated) structure factors;

(iv) multipole refinement against these structure factors to get aspherical scattering factors;

(v) ‘whole-molecule’ aspherical atom refinement of the real crystal structure.

**Figure 1:**
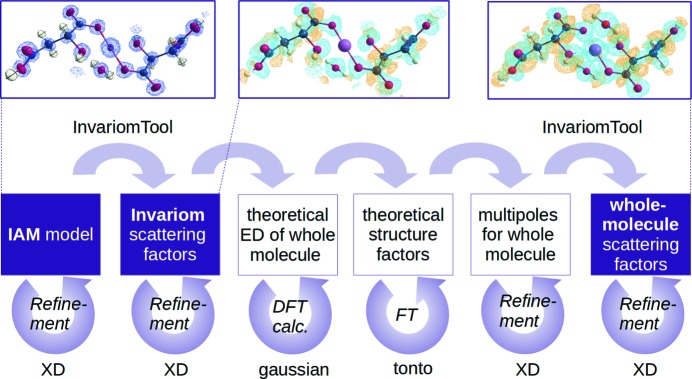
Graphical representation of the ‘whole-molecule’ work flow. Boxes with a purple background represent the models refined against XRD data from the experiment, while the white boxes are purely computational.

The approach is illustrated in Fig. 1 ▸.

The IAM, invariom and ‘whole-molecule’ models were compared for each data set and central metal atom. Figures of merit can be compared directly for all three models since the number of parameters refined is the same; multipole parameters are kept at the values refined against the theoretical data in the latter two refinements.

#### 2.2.1. Metal atoms on special positions   

In half of the structures investigated, atoms are situated on special positions. In these cases, a symmetry operation has to be applied to the asymmetric unit to complete the molecule in question before a quantum-chemical computation of the molecular EDD can be carried out. Although a recent study (Thangavel *et al.*, 2015 ▸) suggested calculation of the crystallographic unit cell, equivalent results are obtained by completing the molecule before continuing with the work flow. For atoms on a special position in a real crystal structure, only those multipoles that agree with the respective local-atomic site symmetry are populated.

#### 2.2.2. Complexes where multiple electronic configurations are possible   

The spin state of a metal atom needs to be taken into account in the calculation of a molecular wave function. Spin states were deduced from ligand field theory (LFT) considerations for all compounds investigated. Since LFT is a rather crude approximation and molecular wave functions are easily accessible *via* DFT computations, energies from the quantum mechanics (QM) calculations of high-spin (hs) and low-spin (ls) states are compared for all nickel and cobalt complexes.

#### 2.2.3. The role of anomalous dispersion   

A factor that can facilitate distinguishing metal atoms by single-crystal XRD is the almost instantaneous interaction between high-energy photons and core electrons, called anomalous dispersion. Anomalous scattering is modelled by element and energy-dependent (but, to a good approximation, resolution independent) real and imaginary anomalous scattering factors 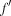 and 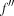 during least-squares refinement. 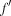 and 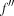 are well known for laboratory experiments with monochromatic X-rays generated from common anode materials. It matters whether a compound is centrosymmetric or not; only for centrosymmetric structures, like the ones studied in this work, does Friedel’s law hold. For noncentrosymmetric structures, the anomalous scattering contribution of an element can make distinguishing, for example, copper and nickel, with their rather different values of 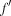 (Prince, 2004 ▸) for copper radiation a lot easier, although for merged data sets, information on anomalous dispersion gets lost^7^. When energy-dispersive X-ray spectroscopy (EDXS) is available, the absorption edges of a particular element can identify unambiguously the elements present in a sample. This can also be achieved with tunable synchrotron radiation when only one single crystal of a given sample is studied. However, X-ray experiments for connectivity determination are not usually accompanied by EDXS measurements, and the use of synchrotron radiation is still an expert domain.

### 2.3. Crystal structures studied   

In this project, 11 pairs of centrosymmetric crystal structures (Zhang, 2007 ▸; Liu, 2007*a* ▸,*b* ▸; Wu *et al.*, 2007 ▸; Liu *et al.*, 2007 ▸; Wang *et al.*, 2005*a* ▸,*b* ▸; Zhu *et al.*, 2003 ▸, 2006 ▸; Ju *et al.*, 2006 ▸; You, 2005*a* ▸,*b* ▸; Chen, 2006 ▸; Wang, 2007 ▸; Zhao, 2007 ▸; Hou, 2007 ▸; Wang & Qiu, 2006 ▸; Sun *et al.*, 2005*a* ▸,*b* ▸; Yang, 2005*a* ▸,*b* ▸; Liu & Zeng, 2006 ▸) from data deposited in the CSD were investigated. Each pair had the same unit cell and compound geometry, but contained different metals as the central atom. In some cases, the reflection data sets differed only by a scale factor^8^; in others, they were from different measurements of the X-ray data, but were isotypic. It should be kept in mind that chances of finding the combination of two distinct compounds with different metals but with very similar unit-cell parameters and atomic positions are small, although in general, isotypism might not be that rare.

### 2.4. Outlook: program availability and usability, practical considerations   

Scientific results obtained with aspherical scattering factors are interesting and have been obtained continuously throughout the last decade by a number of expert users with access to charge-density refinement and analysis programs (including *XD* and *MOPRO*). Despite this, an extensive group of users of the invariom database or competing approaches is lacking.

Fruitful discussions with almost all project leaders involved in developing current small-molecule least-squares refinement programs have shown that implementing useful tools used in conventional structure analysis in charge-density programs, or likewise adding a pseudoatom scattering-factor formalism to existing IAM refinement programs, are both hard to achieve at a quality standard that small-molecule crystallographers are used to. This is primarily due to fundamental design decisions made earlier. However, experience gained over the last decade has also shown that there are no fundamental hurdles that forbid using aspherical scattering factors more frequently. Our focus so far has been to obtain novel scientific results that can be achieved by moving to aspherical scattering factors, rather than to provide a black-box tool with the robustness and ease-of-use of software such as *SHELXL* (Sheldrick, 2015*b* ▸) [in combination with graphical user interfaces like *ShelXle* (Hübschle *et al.*, 2011 ▸) or *OLEX2* (Dolomanov *et al.*, 2009 ▸)].

We think that the combination of aspherical scattering factors, possibly with estimated hydrogen atomic displacements and refined hydrogen positions, as well as subsequent property calculation of dipole moments and electrostatic potentials, is an important part of the future of small-molecule XRD. Users can achieve higher accuracy without the need to increase the resolution of a diffraction experiment beyond what can be reached with copper radiation, potentially also without expert knowledge. While research aimed at the development of aspherical atom refinement programs and the measurement of high-resolution structures remains fundamentally important in charge-density research, this should still leave room to address the need for a least-squares implementation of tabulated aspherical scattering factors, to provide a process that is as robust and covers the same functionality as, for example, *SHELXL*. Efforts to implement an aspherical scattering factor model into *SHELXL* have been initiated.

## 3. Results and discussion   

Four of the 11 cases studied are discussed as examples here, while the other structures are discussed in a similar way in the supporting information and included in the overview at the end of this section.

### 3.1. Pair (1): diaquabis(malato-κ^2^ *O* ^1^,*O* ^2^)nickel(II)/copper(II)   

**Figure 2:**
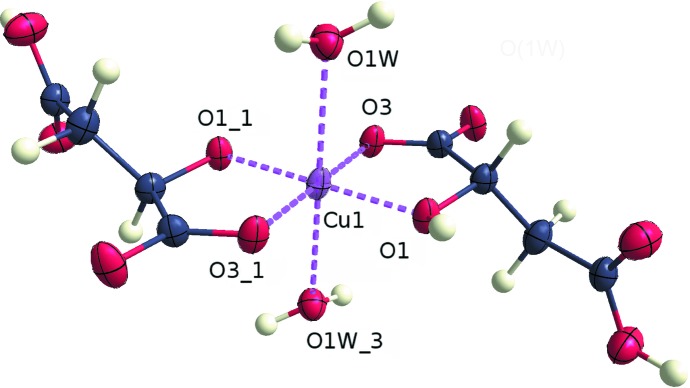
*ORTEP*-type (Burnett & Johnson, 1996 ▸) displacement ellipsoid plot at a probability of 50% of pair (1) with Cu after refinement of the whole-molecule scattering factors against data set (1*a*). The symmetry-equivalent ligand is also displayed. The figure was generated with *molecoolQt* (Hübschle & Dittrich, 2011 ▸).

In this case of diaquabis(malato-κ^2^ *O* ^1^,*O* ^2^) complexes, the central metal atoms were nickel(II) (Liu, 2007*a* ▸) and copper(II) (Zhang, 2007 ▸) (Table 1 ▸). The metal atom lies on an inversion centre, so the asymmetric unit contains only one ligand and one water molecule. The two malate ligands constitute the equatorial plane. Each coordinates to the metal atom *via* the O1 and O3 atoms. Additionally, two water molecules coordinate in the axial positions with longer O—*M* distances. Atoms O1 and O3 are situated 1.9556 (10) and 1.9123 (10) Å from the metal atom, while the water O atom is 2.5192 (11) Å away^9^. An *ORTEP* plot (Burnett & Johnson, 1996 ▸) showing the atom numbering for the copper(II) complex, *i.e.* (1*a*), is depicted in Fig. 2 ▸.

Data set (1*a*) (Zhang, 2007 ▸) contains reflections up to a higher resolution and an intensity that is consistently 1.094 times that of the reflections in data set (1*b*) (Liu, 2007*a* ▸). The unit-cell parameters are identical, although the number of reflections used for the cell determination is different according to the deposited crystallographic information. Whereas data set (1*a*) was supposedly measured at 293 (2) K, data set (1*b*) is stated to have been measured at 298 (2) K. Nonetheless, the reflection data are the same. Because of this, the two structural models differed only by the identity of the metal atom and the correct metal atom was all that had to be identified.

#### 3.1.1. Chemical reasoning   

As discussed previously, the coordination geometry of the complex is an axially elongated octahedron. This provides a strong argument for the metal to be copper(II), as it has a *d* ^9^ electron configuration, which unlike the *d* ^8^ configuration for nickel(II), profits energetically from Jahn–Teller (JT) splitting of the orbitals. Therefore, basic orbital considerations already suggest copper(II) as the correct metal.

#### 3.1.2. Refinement results   

**Figure 3:**
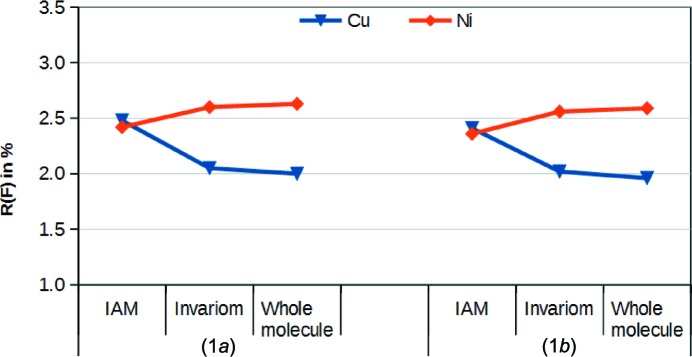
Comparison of *R*(*F*) values for the refinements of the different metal atoms with different EDD models against the two data sets for pair (1).

This structure is a typical example where in the IAM the heavier metal provided the worst fit to the reflection data (see Table 2 ▸). However, upon invariom modelling, the fit for copper(II) improves. The gap between nickel(II) and copper(II) increases still further with aspherical atom modelling of the whole molecule. Fig. 3 ▸ shows that, with increasing model quality, the ability of nickel(II) to fit the data gets progressively worse compared to copper(II). These observations therefore provide compelling evidence that copper(II) is the correct metal.

### 3.2. Pair (4): bis(2-amino­pyridine)­di­benzoato­cobalt(II)/nickel(II)   

**Figure 4:**
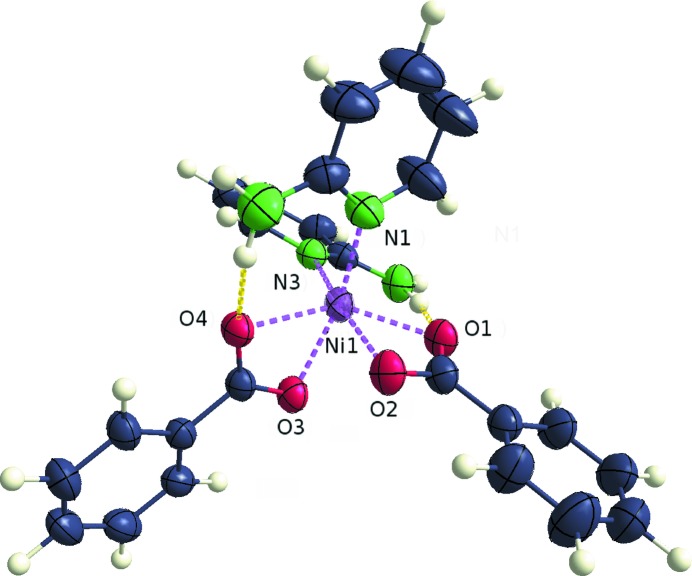
*ORTEP*-type displacement ellipsoid plot at a probability of 50% for pair (4), with nickel(II) as the central atom after refinement of the ‘whole-molecule’ scattering factors against data set (4*a*).

In octahedral bis(2-amino­pyridine)­di­benzoato­metal(II) com­plex (4), shown for the refinement using nickel(II) in Fig. 4 ▸, the pyridine ligands are in a *cis* configuration and the negative charges of the two anionic benzoate ligands are distributed over all of the coordinating O atoms. The crystal structure could contain either nickel(II) or cobalt(II) as the central atom, as data sets (4*a*) (Zhu *et al.*, 2003 ▸) and (4*b*) (Ju *et al.*, 2006 ▸) (Table 3 ▸) are the same apart from multiplication by a factor^10^. The unit cells are identical, although a different number of reflections was reported to have been used for the cell determinations [*i.e.* 2530 for (4*b*) and 19350 for (4*a*)].

#### 3.2.1. Chemical reasoning and spin state   

In an octahedral environment, both metals, *i.e.* cobalt(II) and nickel(II), are similarly plausible. Spin states and JT distortions are discussed for the structures of pair (3) (see the supporting information), where the same two metals were considered in an octahedral complex. Concerning the possibility of a JT distortion, the displacement ellipsoids show no special elongation in the direction of the coordinate bonds. However, O3 is farther away from the metal atom than O2, despite the fact that they are ostensibly chemically equivalent. Similarly, *M*—O1 is longer than *M*—O4 (see Table 4 ▸ and Fig. 4 ▸). The bonds to O2 and O3 are each *trans* to a donating N atom and are on average longer than those *trans* to another O atom. In general, the arrangement is such that the opposing O2/N3 pair display, in both cases, the closest distance to the centre among the equivalent atoms. Hence, a JT deformation is possible, but, in this case, the JT effect would have to be dynamic. The disparity in the bond lengths could be caused by a slight inequality in the tilting of the two benzoate ligands, which would explain that the difference between the bond lengths involving O1 and O4 is greater than their deviation from the distance of O2 to the central atom. A comparative investigation using the diffraction data was required since there was no clear distinction between the identities of the metals on the basis of the atomic coordinates.

#### 3.2.2. Refinement results   

**Figure 5:**
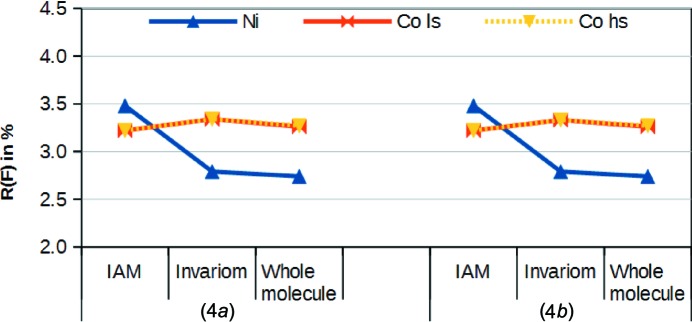
Comparison of *R*(*F*) values for the refinements of the different metal atoms with different EDD models against the two data sets for pair (4).

In the IAM refinements, the usual pattern that the lighter atom yielded a better fit to the data emerged. As shown in Fig. 5 ▸, this changes considerably for the invariom refinement. *R*(*F*) dropped from 3.48 to 2.79% for nickel(II), while it increased from 3.22 to 3.34% for cobalt(II). Both models again profit from the inclusion of aspherical modelling around the central atoms. The improved ‘whole-molecule’ models also showed a clearly better fit for nickel(II) than for cobalt(II) in either spin state (Table 5 ▸), once again providing clear evidence that in this structure the correct metal is nickel(II).

### 3.3. Pairs (8) and (9): bis[4-bromo-2-(cyclohexylimino­methyl)phenolato]cobalt(II)/nickel(II)/copper(II)/zinc(II)   

In this case, four isotypic bis[4-bromo-2-(cyclohexyl­iminomethyl)phenolato]- complexes of the 3*d* metals cobalt(II) (Wang & Qiu, 2006 ▸), nickel(II) (Sun *et al.*, 2005*b* ▸), copper(II) (Yang, 2005*a* ▸) and zinc(II) (You, 2005*a* ▸) (Table 6 ▸) were investigated. Their single-crystal XRD data sets were each different from one another. In contrast, the unit-cell constants for data sets (8*a*) and (8*b*) are identical, while for data sets (9*a*) and (9*b*) they differ by only by 0.2% (see Table 7 ▸) and can thus also be considered to be the same (Herbstein, 2000 ▸).

#### 3.3.1. Chemical reasoning   

The complexes adopt a tetrahedral coordination geometry and crystallize in the space group *Pbca*. At first sight, it seems most unlikely that all four metal ions would crystallize with identical coordination geometries, in view of the fact that their electronic structures differ by up to three electrons.

While tetrahedral coordination environments are common for zinc(II), copper(II) usually forms JT-distorted octahedra or square-planar complexes (Hoffmann & Goslar, 1982 ▸) if the ligands impose a weak ligand field that would induce only a small ligand-field splitting. However, strong-field ligands, generating large ligand-field splittings, as well as bulky ligands, can lead to more tetrahedral arrangements. Amines are strong-field ligands, while the hydroxide anion is weak. Therefore, no clear conclusions can be derived concerning the likely coordination environment of copper(II) from ligand field theory.

There is an example of a copper(II) complex with an extremely large ligand (Costamagna *et al.*, 1998 ▸) having bromide ions that lie reasonably distant from the metal. In this structure, a fifth and sixth coordinating ligand complete a JT-distorted octahedron, where the arrangement of the inner ligating atoms resembles a tetrahedron. Regular tetrahedra of copper(II) complexes are not stable (Hoffmann & Goslar, 1982 ▸) due to the JT effect of the *t*_2_ orbitals. However, from the bond angles listed in Table 8 ▸, the tetrahedral coordination is far from perfect in complexes (8) and (9). No additional contacts that could involve coordination are found at longer distances in the structure model; two H atoms lie 3.02 and 3.71 Å away, the closer belonging to the cyclohexyl group. Furthermore, Schiff base ligands are known to form almost tetrahedral coordination geometries with copper(II) (Cinčić & Kaitner, 2011 ▸), hence chemical reasoning alone could not exclude copper(II) as the correct central metal atom in this case.

#### 3.3.2. Spin state   

Cobalt(II) and nickel(II) can be either high-spin in tetrahedral complexes or low-spin in a square-planar geometry. DFT results show a preference for the high-spin state for both cobalt(II) and nickel(II) in single-point energy calculations at experimental molecular geometries.

#### 3.3.3. Refinement results   

Refinements were performed with all four metals for each of the four data sets. The fit for cobalt(II) was the worst in each case, and nickel(II) did not fit well either. Copper(II) and zinc(II) yielded the best residuals. A finding with more general validity was that the better fit of the heavier element in the IAM was an indication for incorrectness of the lighter element, also taking into account the common oxidation state.

After aspherical modelling of the ligands, the cobalt(II) and nickel(II) models did not improve, but those with copper(II) and zinc(II) did. The zinc(II) model improved the most and, for data sets (8*a*) and (8*b*), this led to a lower *R*(*F*) value for zinc(II) than for copper(II). This contrasts sharply with the IAM results, in which both metals fitted almost equally well. For data sets (9*a*) and (9*b*), the results for zinc(II) that were initially worse became almost as good as those for copper(II) following aspherical atom modelling.

**Figure 6:**
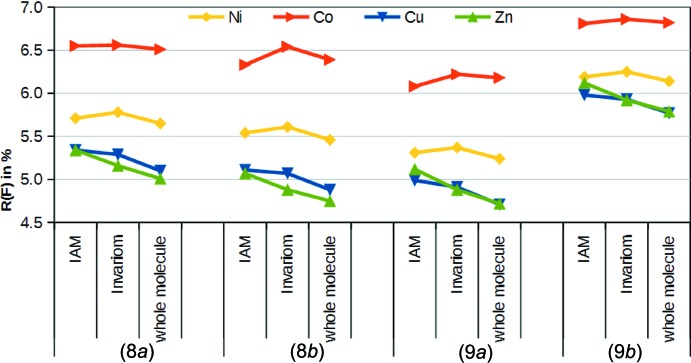
Comparisons of *R*(*F*) values for the refinements of the different metal atoms with different EDD models against the two data sets of pairs (8) and (9).

Inclusion of multipoles for the central atom in the models led to better modelling of the EDD throughout (Fig. 6 ▸). For data sets (8*a*) and (8*b*), zinc(II) again produced the best results. Data sets (9*a*) and (9*b*) were found to differentiate between copper(II) and zinc(II) less effectively. Indeed, the results for (9*a*) and (9*b*) were reasonably similar to those for (8*a*) and (8*b*). However, even taking into account the slightly different unit-cell constants, it is likely that all of the four structures are from the same complex.

#### 3.3.4. Isotypism and geometrical aspects   

As shown in Table 9 ▸, bond lengths involving the metal atom do not differ significantly. Only very few isotypic Schiff base complexes are reported in the literature (Amirnasr *et al.*, 2002 ▸; Cinčić & Kaitner, 2011 ▸; Sacconi & Ciampolini, 1964 ▸), with none found that contain zinc or copper. This already suggests that it is most unlikely that the copper(II) and zinc(II) complexes (9*a*) and (9*b*) are isotypic. However, as two isostructural complexes with only minor changes in geometry (around 0.01 Å for bonds to nitrogen; Amirnasr *et al.*, 2002 ▸) were reported for metals differing by two (cobalt and copper; Amirnasr *et al.*, 2002 ▸) and three electrons, respectively (cobalt and zinc; Cinčić & Kaitner, 2011 ▸), copper(II) cannot be excluded as a possibility with complete certainty here.

#### 3.3.5. Energetic considerations   

In order to better distinguish copper(II) or zinc(II) in (9), the structures of the two complexes were optimized using the same DFT method as that used for the single-point calculation which provided the molecular EDD. The gain in energy upon geometry relaxation was greater for copper(II) compared to both the starting geometries from structures (8) and (9) (Table 10 ▸ 11). This independent quantum-chemical information confirms that zinc(II) is the correct atom, in agreement with the refinement of the invariom models against the XRD data.

#### 3.3.6. Refinement of metal occupancies   

An alternative tool to obtain indications for distinguishing cobalt(II), nickel(II), copper(II) and zinc(II) would be to refine an occupancy of the central atom as an additional free variable in *SHELXL*. This approach is certainly easier to carry out than aspherical atom refinements, since it relies on the IAM. The results are in full agreement with our earlier findings: cobalt(II) has an average occupancy in excess of 111 (1)%, with the occupancy decreasing *via* nickel(II) with 106 (1)% and copper with 100 (1)% to zinc(II) with 98 (1)% (average values of all four data sets). Cobalt(II) and nickel(II) can again be excluded, copper(II) and zinc(II) can, however, not be distinguished well enough this way, especially taking into account that dative bonding might, sometimes noticeably (Dittrich *et al.*, 2015 ▸), reduce the EDD around the central atom. We therefore think that this methodology can only provide first indications, but not the certainty that is desirable.

#### 3.3.7. Conclusion   

In summary, refinements show that structures (8*a*) and (8*b*) contain zinc(II) and definitely not cobalt(II) or nickel(II) as the central metal atom. For structures (9*a*) and (9*b*), the data quality was not sufficiently high to dis­tin­guish unambiguously between copper(II) and zinc(II). However, by com­paring figures of merit from the refinements, together with unit-cell parameters, bond lengths and angles, it is very likely that both data sets for (9) contain the same element. This also agrees with subsequent QM calculations. Zinc(II) is therefore most likely to be the central metal atom in all four structures.

### 3.4. A summary of all the pairs of structures studied   

The methodology and results for the other structural pairs investigated were similar and a detailed description is given in the supporting information. The final conclusions for each of the structures, together with other relevant information, are given in Table 11 ▸. In seven cases, the identity of the central metal atom was successfully established from the deposited single-crystal XRD data. Limitations of the invariom-like approach become apparent from an examination of the four structures (8)–(11). We find – despite the fact that data were collected at room temperature in all cases – that data quality does not necessarily have to be excellent in order to achieve satisfactory results. However, with very noisy data sets, the results might not be precise enough and require further chemical considerations, as in the case of pair (10); the results from the fit to the XRD data suggest that the metal is copper(II) for the tetrahedral complex. As the data quality is low in this case, this study mostly serves as an indicator for further inquiries.

Case (11) demonstrates the limitations of the method when dealing with disordered structures, although these are technically possible (Dittrich *et al.*, 2016 ▸); the disordered structure (11) and its diffraction data cannot be used to determine unambiguously the elements present.

Finally, in the quartet of similar structures [pairs (8) and (9)], two of the metals, *i.e.* cobalt(II) and nickel(II), could be excluded simply by evaluating the fit of the models to the data. For two of the four structures, *i.e.* pair (8), zinc(II) could be identified unambiguously as the correct central metal atom. However, the identity of the remaining two metals was derived from energy considerations, *i.e.* based on changes in energy upon relaxation of the crystal geometry. These computations, together with previous knowledge of the chemistry of copper complexes and their isotypic behaviour, pointed to zinc(II) as the most likely candidate for the central atom in pair (9). Data quality was the limiting factor here, as was also the case for example (10).

Overall, it was demonstrated that invariom modelling of the ligand environment only (omitting the asphericity of the metal atom) is a helpful tool for identifying the correct metal atom in structures of coordination complexes where the available X-ray data are at least of moderate quality and resolution. In contrast, the information from IAM refinement was usually insufficient to determine the correct identity of the central metal in the complex. Generating and using aspherical scattering factors for the whole molecule, including the metal centre, can further increase the quality of the model and its distinguishing power. However, this is not always mandatory for successful identification of the metal atom. Model quality is already sufficiently improved by describing the ligand(s) using aspherical scattering factors only and this is because of the change in the overall scale factor and the better deconvolution of thermal motion and EDD.

Therefore, future investigations of potentially fraudulent pairs of structures could initially employ scattering factors from the invariom database for the ligand, assuming full charge transfer between the metal and ligand environments (Nelyubina & Lyssenko, 2015 ▸). Treatment of the whole molecule with aspherical scattering factors would be worth the extra effort only in cases where the results from the simpler model are not sufficiently convincing. An example of such a case, in which almost no improvement was observed upon invariom modelling is case (6*b*). In most cases, however, invariom modelling alone should improve the model enough to distinguish between the two metal atoms.

Coincidentally, all examples were measured with Mo *K*α radiation. Similar results can be obtained with other common anode materials like copper, gallium or silver and their radiation.

## 4. Conclusion   

Aspherical-atom refinement with conventional data sets is now possible for coordination compounds. New model compounds and those already present in the invariom database have been geometry-optimized using the Minnesota density functional M06, in combination with Ahlrichs’ def2TZVP all-electron basis set, increasing the range to include all elements up to bromine (krypton). This method/basis set combination has been used successfully for a series of compounds containing 3*d* transition metals and permits the treatment of all the elements present at the same level of theory. To highlight current progress, we have re-investigated a number of pairs of published structures, where the element-type assignment of the metal was unclear, and where duplicates were published based on the same sets of X-ray data or with different data sets but the same unit-cell parameters. We show that aspherical scattering factors permit identification of the correct structure without any further chemical or spectroscopic evidence using the originally deposited diffraction data. These data were usually of conventional resolution (*d* ≤ 0.84 Å) and measured at room temperature. An interesting aspect is that distinguishing the 3*d* metal atoms did not usually require the modelling of the asphericity of the metal atom itself.

The ability to improve and possibly correct results from earlier experiments is an obvious advantage of (aspherical-atom refinement in) single-crystal XRD. As this technique relies on the availability of the original X-ray data, its success with these problem structures highlights the importance of depositing the originally measured data.

## 5. Related literature   

References cited in the supporting information include: El Haouzi *et al.* (1996 ▸), Frisch *et al.* (2013 ▸), Hollemann *et al.* (2007 ▸), Hübschle *et al.* (2007 ▸), Kitajima *et al.* (1990 ▸), Müller *et al.* (2006 ▸) and Sheldrick *et al.* (2015*a* ▸).

## Supplementary Material

Supporting information. DOI: 10.1107/S2052520617010745/bm5092sup1.pdf

## Figures and Tables

**Table 1:** Structural formula and selected crystallographic and chemical information of pair (1) 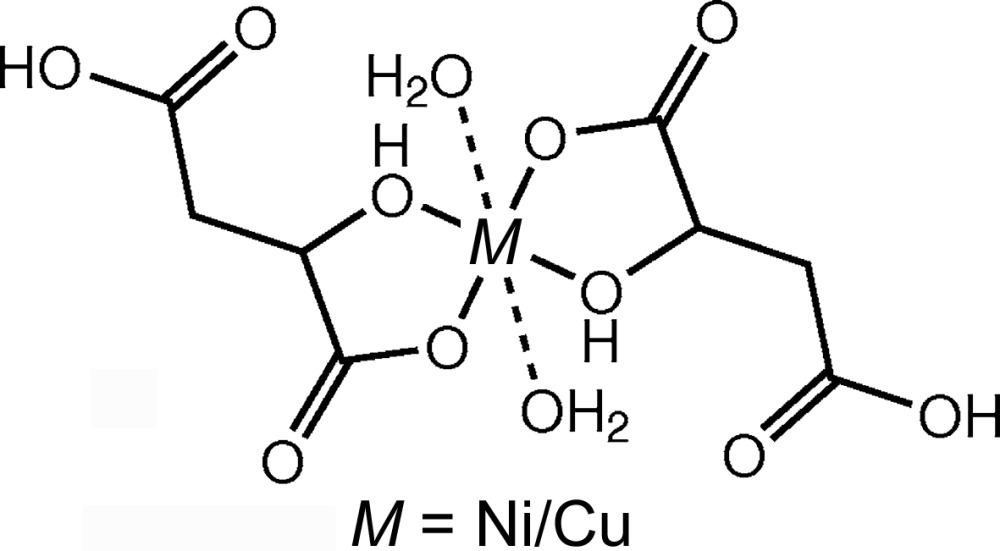

	Data set (1*a*)	Data set (1*b*)
Literature	Zhang (2007)	Liu (2007*a*)
IUCr code	dn2151	hb2526
CSD code	BESNOC01	XILXIA
CCDC No.	647183	664185
Space group	*P*2_1_/*c*	
Peculiarity	*M* on special position ( 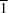 )	
Coordination geometry	Square planar	
Metal ion	Cu^2+^	Ni^2+^
Electron configuration	[Ar]4*s* ^0^3*d* ^9^	[Ar]4*s* ^0^3*d* ^8^
Spin multiplicity	2	3

**Table 2:** Selected computational and refinement results for pair (1) *E*(M06) is the energy obtained with the M06 density functional in the unrestricted formalism. The correct result with the lower *R*(*F*) is highlighted in bold.

	Cu^2+^	Ni^2+^
*E*(M06) crystal geometry (a.u.)	−2856.475	−2724.308
*R*(*F*) (%) against theoretical data	0.47	0.47
*R*(*F*) (%) whole mol­ecule data set (1*a*)	**2.00**	2.63
*R*(*F*) (%) whole mol­ecule data set (1*b*)	**1.96**	2.59

**Table 3:** Structural formula and selected crystallographic and chemical information of pair (4) 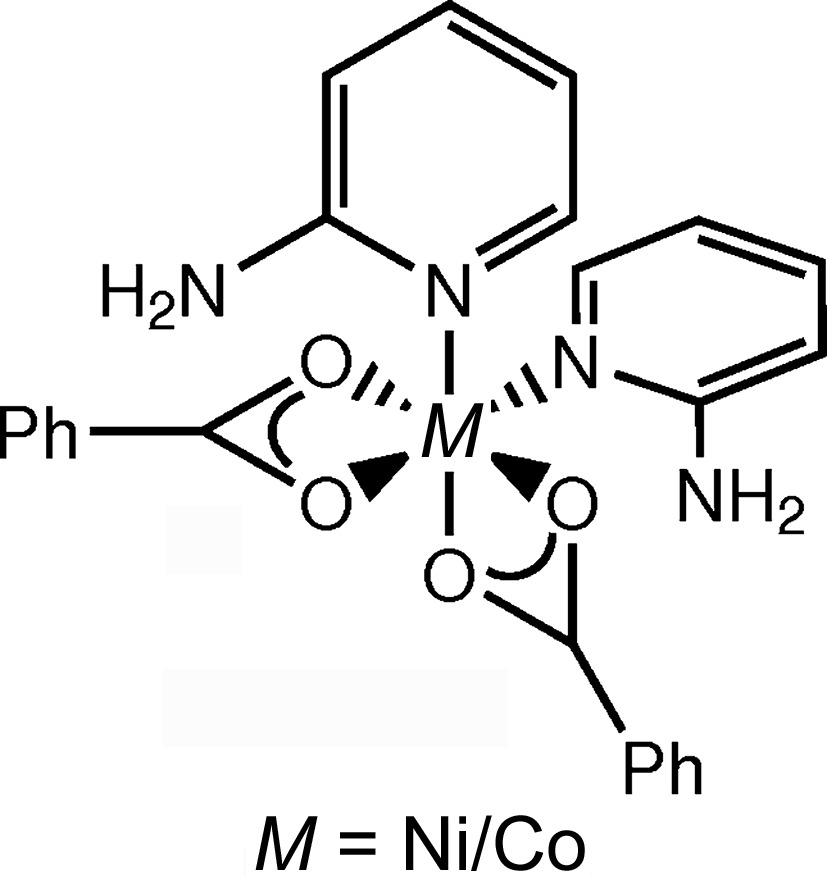

	Data set (4*a*)	Data set (4*b*)
Literature	Zhu *et al.* (2003)	Ju *et al.* (2006)
IUCr code	lh6101	hb2030
CSD code	OLOJOO	IDOKOC
CCDC No.	225650	608593
Space group	*C*2/*c*	
Peculiarity	–	
Coordination geometry	Distorted octa­hedral	
Metal ion	Ni^2+^	Co^2+^
Electron configuration	[Ar]4*s* ^0^3*d* ^8^	[Ar]4*s* ^0^3*d* ^7^
Spin multiplicity	3	2, 4

**Table 4:** Selected bond lengths (Å) from the final structure of pair (4)

Ni1—O1	2.1279 (9)	Ni1—N3	2.0595 (11)
Ni1—O4	2.0733 (8)	Ni1—O2	2.1150 (10)
Ni1—N1	2.0625 (12)	Ni1—O3	2.1760 (9)

**Table 5:** Selected computational and refinement results for pair (4) *E*(M06) is the energy from the calculation with the M06 density functional. The correct result with the lower *R*(*F*) is highlighted in bold.

	Ni	Co (high spin)	Co (low spin)
*E*(M06) crystal geometry (a.u.)	−2955.783	**−2830.223**	−2830.202
*R*(*F*) (%) against theoretical data	0.45	0.48	0.46
*R*(*F*) (%) whole mol­ecule data set (4*a*)	**2.74**	3.27	3.26
*R*(*F*) (%) whole mol­ecule data set (4*b*)	**2.74**	3.27	3.26

**Table 6:** Structural formula and selected crystallographic and chemical information of pairs (8) and (9) 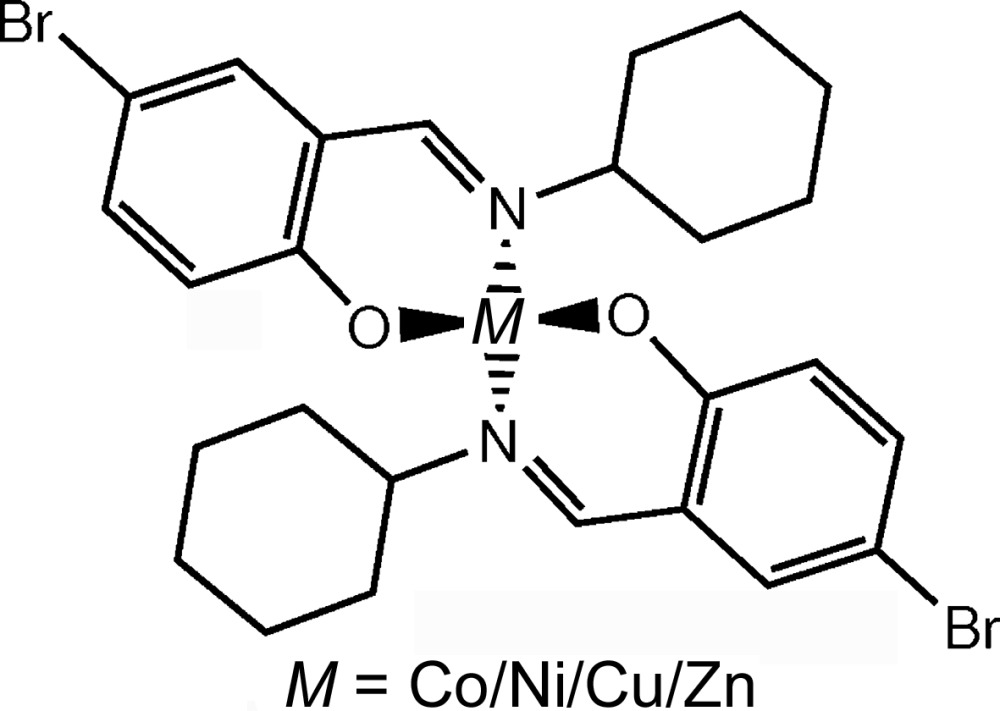

Data set	(8*a*)	(8*b*)	(9*c*)	(9*d*)
Literature	Sun *et al.* (2005*b*)	Wang & Qiu (2006)	Yang (2005*a*)	You (2005*a*)
IUCr code	ci6575	ci2197	ob6467	ci6692
CSD code	FETLEW01	PESSEM	FAYMOI	YAYXUS
CCDC No.	274365	629307	263535	281782
Space group	*Pbca*			
Peculiarity	None			
Coordination geometry	Tetra­hedral			
Metal ion	Ni^2+^	Co^2+^	Cu^2+^	Zn^2+^
Spin multiplicity	3	2,4	2	1

**Table 7:** Unit-cell parameters (Å) for pairs (8) and (9) Δ is the maximal absolute difference between the individual vectors.

Unit cell	*a*	*b*	*c*
Data set (8*a*)	14.979 (3)	13.609 (3)	25.164 (5)
Data set (8*b*)	14.9790 (10)	13.6090 (10)	25.1640 (10)
Data set (9*a*)	14.9960 (10)	13.5970 (10)	25.156 (2)
Data set (9*b*)	14.9830 (10)	13.5870 (10)	25.143 (2)
Max. Δ	0.017	0.022	0.021
Max. Δ (%)	0.1	0.2	0.1

**Table 8:** Bond angles (°) for pairs (8) and (9) from the zinc model compound using aspherical scattering factors for the whole mol­ecule

	O1—Zn—O2	N1—Zn—N2	O1—Zn—N1	O1—Zn—N2	O2—Zn—N1	O2—Zn—N2
Data set (8*a*)	119.65 (11)	122.46 (12)	93.74 (11)	113.76 (12)	113.81 (12)	95.48 (11)
Data set (8*b*)	119.86 (10)	122.42 (11)	93.84 (10)	113.71 (10)	113.75 (11)	95.36 (10)
Data set (9*a*)	119.94 (10)	122.40 (10)	93.66 (10)	113.85 (10)	113.74 (10)	95.36 (10)
Data set (9*b*)	119.82 (13)	122.47 (13)	93.72 (13)	113.93 (13)	113.70 (13)	95.30 (13)

**Table 9:** Bond lengths (Å) for the pairs (8) and (9) from the zinc model using aspherical scattering factors for the whole mol­ecule

	Zn1—O1	Zn1—O2	Zn1—N1	Zn1—N2
Data set (8*a*)	1.914 (3)	1.913 (3)	2.028 (3)	2.023 (3)
Data set (8*b*)	1.913 (2)	1.915 (2)	2.025 (3)	2.026 (3)
Data set (9*a*)	1.913 (2)	1.916 (2)	2.032 (3)	2.031 (3)
Data set (9*b*)	1.914 (3)	1.914 (3)	2.029 (3)	2.033 (3)

**Table 10:** Comparison of the single-point SCF energies (in Hartree) and those after geometry optimization for pairs (8) and (9) with copper and zinc

	Pair (8)		Pair (9)	
	Cu	Zn	Cu	Zn
Starting geometry	−8057.0633	−8195.9862	−8057.0629	−8195.985
Optimized geometry	−8057.0819	−8195.9933	−8057.0819	−8195.9933
Ratio	1.00000230	1.00000087	1.00000235	1.00000093
Change (%)	0.00023	0.00009	0.00023	0.00009
Difference (a.u.)	−0.0185	−0.0071	−0.0189	−0.0076
Change (kJ mol^−1^)	−48.7	−18.6	−49.7	−20.0

**Table 11:** A summary of all the pairs of structures studied

Pair	Coordination geometry	Reference	Metal	Diffraction data	Conclusion	Remarks
(1)	Square planar	Zhang (2007 ▸)	Cu	The same	Cu correct	*M* on special position ( 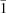 )
		Liu (2007*a* ▸)	Ni			
(2)	Octa­hedral	Wu *et al.* (2007 ▸)	Ni	The same	Cu correct	Three water molecules in the asymmetric unit
		Liu *et al.* (2007 ▸)	Cu			
(3)	Octa­hedral	Wang *et al.* (2005*b* ▸)	Ni	Not the same	Ni correct	Was also compared with Cu
		Wang *et al.* (2005*a* ▸)	Co			Four water molecules in the asymmetric unit
(4)	Octa­hedral	Zhu *et al.* (2003 ▸)	Ni	The same	Ni correct	
		Ju *et al.* (2006 ▸)	Co			
(5)	Square planar	You (2005*b* ▸)	Ni	Not the same	Ni correct	*M* and C10 on special positions
		Chen (2006 ▸)	Co			Space group *A*2_1_ *am*
(6)	Square planar	Wang (2007 ▸)	Ni	The same	Cu correct	*M* on special position ( 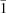 )
		Liu (2007*b* ▸)	Cu			
(7)	Square planar	Zhao (2007 ▸)	Ni	Not the same	Cu correct	*M* on special position ( 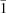 )
		Hou (2007 ▸)	Cu			
(8)	Tetra­hedral	Sun *et al.* (2005*b* ▸)	Ni	Not the same	Most likely Zn	Isotypic with pair (9)
		Wang & Qiu (2006 ▸)	Co		Most likely Zn	
(9)	Tetra­hedral	Yang (2005*a* ▸)	Cu	Not the same	Cu or Zn	Isotypic with pair (8)
		You (2005*a* ▸)	Zn		Cu or Zn	
(10)	Tetra­hedral	Yang (2005*b* ▸)	Cu	The same	Cu fits better	Average data quality, different resolutions
		Liu & Zeng (2006 ▸)	Ni			*M* on special position (2)
(11)	Square planar	Sun *et al.* (2005*a* ▸)	Ni	Not the same	Ambiguous	Identification difficult due to ligand disorder (hinting at Cu)
		Zhu *et al.* (2006 ▸)	Cu			

## Footnotes

1 Such comparisons of different data sets can also reveal when two apparently isomorphous structures have reflection data deviating only by a scale factor, implying a linear correlation if both data sets are plotted against each other.

2 Four scattering-factor databases currently exist: the ‘supramolecular-synthon-based fragments approach’ (SBFA; Hathwar *et al.*, 2011 ▸), the ‘experimental library multipolar atom model’ (ELMAM2; Zarychta *et al.*, 2007 ▸; Domagala *et al.*, 2012 ▸) (both based on high-resolution experiments), the ‘generalized invariom database’ (GID; Dittrich *et al.*, 2006*a* ▸, 2013 ▸) and the ‘University at Buffalo Databank’ (UBDB2011; Dominiak *et al.*, 2007 ▸; Jarzembska & Dominiak, 2012 ▸) [the latter two are based on theoretical density functional theory (DFT) computations]. All four rely on the established Hansen–Coppens multipole model (Hansen & Coppens, 1978 ▸) and can be used successfully to improve the accuracy and precision of least-squares structure refinements.

3 The Hansen–Coppens multipole model can be seen as a (minor) modification of R. F. Stewart’s generalized scattering-factor model (Stewart, 1976 ▸) with additional κ-screening parameters. The implementation of this earlier model in *VALRAY* (Stewart *et al.*, 1998 ▸) does not allow for local-atomic coordinate systems and is frequently considered to be not as user friendly as the one of the Hansen–Coppens variety in the successor programs of *MOLLY* (Hansen, 1978 ▸), *e.g.* *XD2006* (Volkov *et al.*, 2006 ▸), *MOPRO* (Guillot *et al.*, 2001 ▸; Jelsch *et al.*, 2005 ▸) and *WinXpro* (Stash & Tsirelson, 2002 ▸).

4 We have also studied 4*d* metals and obtained similar model-related improvements in the fit to the diffraction data than for the cases studied here (results not shown), although one then probably has to use a smaller all-electron basis set, *e.g.* SVP. For 5*d* metal complexes or those containing lanthanides or even actinides, *i.e.* elements with an increasing number of core electrons, the suitability of valence-only scattering factors is diminishing (Stevens & Coppens, 1976 ▸), as will the improvements seen from moving from IAM to aspherical scattering factors.

5 After changing the basis set to one covering bromine also, all chlorine- and fluorine-containing compounds were added to the database with bromine as a substituent.

6 Here, dative bonding (Haaland, 1989 ▸) often leads to changes in bond lengths and thus the value of the bond-distinguishing parameter of the invariom model. However, this does not usually lead to significant redistributions of EDD from the metal atom to the ligand environment — the ligand EDD remains conserved to a large degree.

7 For the convenience of the reader, values are provided for correcting anomalous dispersion when using copper radiation. These are  = −2.3653 and  = 3.6143 *e* for Co, −3.0029 and  = 0.5091 *e* for Ni,  = −1.9646 and  = 0.5888 *e* for Cu, and  = −1.5491 and  = 0.6778 *e* for Zn.

8 Since the aim of our study was not to identify fraudulent behaviour, but to show that aspherical scattering factors are useful in deciding which metal atom is most likely to be correct, the reasons that led to wrong element assignment will not be speculated upon.

9 Distances were taken from the final converged model of the copper-containing molecule refined against data set **a**.

10 This probably results from depositing the same experimental data set after refinements with different metals and therefore different scale factors *k* . The number of reflections for the refinement was the same in both cases, and the only difference is that each reflection intensity of data set (4*a*) is 1.083 times more intense than in data set (4*b*).

11 Use of the data sets (8*b*) and (9*b*), rather than (8*a*) and (9*a*) was attempted to provide input geometries for the single point computation. This yields smaller differences between data pairs (8) and (9).
