# Supplementary material for: Using invariom modelling to distinguish correct and incorrect central atoms in ‘duplicate structures’ with neighbouring 3d elements
Source: Acta Crystallogr B Struct Sci Cryst Eng Mater. 2017 Sep 29;73(Pt 5):794–804. doi: 10.1107/S2052520617010745 (PMC5628397; doi:10.1107/S2052520617010745)
Supplement: Supplementary file 1 [file b-73-00794-sup1.pdf]

STRUCTURAL SCIENCE
CRYSTAL ENGINEERING
MATERIALS

Volume 73 (2017)

Supporting information for article:

Using invariom modelling to distinguish correct and incorrect central atoms in `duplicate structures' with neighbouring 3*d* elements

Claudia M. Wandtke, Matthias Weil, Jim Simpson and Birger Dittrich

1. Supplementary information

1.1. Procedures used in studying the coordination compounds

Each of the eleven cases studied was treated using the same procedures.

File setup Initially crystallographic information (CIF) files and structure factors were downloaded from the CSD, and instruction files for SHELXL-2014 (Sheldrick, 2015*a*) were generated with PLATON (Spek, 2009). The structure factors were then converted into the SHELX HKLF 4 format by the utility program FCF2HKL.[1] If not mentioned otherwise, the XRD data were collected at room temperature with molybdenum Kα radiation. Usually the reflection files excluded systematically absent reflections and contained negative intensities.

SHELXL **IAM refinement** The IAM refinements were repeated on the complexes with each of the metals identified in the original publications. Scattering factors for neutral atoms were used in all cases. For selected structures ionic scattering factors were applied by manually copying the coefficients from International Tables for Crystallography (Prince, 2004). Small changes in the residual factors were observed, but although these tended to improve for the correct metal atom, the correct metal atom could not be identified with certainty. In each case the weighting scheme was adjusted until convergence was reached.

XD **IAM refinement** The transition to the XD program package (Volkov *et al.*, 2006) (version 6.03) was facilitated by the subroutine XDINI within the program. As shown in the first box of Figure 1, the IAM refinement was repeated for each metal in the set using the subprogram XDLSM. After fixing selected atomic coordinates to accommodate the floating origins in space groups with only translational symmetry

[1] written by G. M. Sheldrick, Georg-August University Göttingen, 2014.

elements in one direction, the model was refined against F. Moreover, the ADPs of atoms on special positions were constrained to reflect the site symmetry. Hydrogen atoms were treated using the riding atom model with their displacement parameters fixed to 1.2 and 1.5 times the parent atom's U_{eq}.[2] Unlike refinement with SHELXL, the weighting scheme was not refined with XD but kept purely statistical ($w = \frac{1}{\sigma^2}$), because adjustment of the weights should be determined following correct assignments of the elements in the structure. In this instance the identity of the elements in the structure is being investigated. Hence the decision not to adjust the weights is sensible in this situation. This was also supported by the unusually high second weighting factors in the SHELXL refinements.

The element name, number of valence electrons in the *d*-orbitals and the dispersion corrections all needed manual adjustment in the files read by XD, in order to change the metal atom for the refinements with this program. In all the XD refinements discussed in this chapter the scattering factors of the ionized metal were used and valence electrons as well as names were set accordingly in the appropriate files.

Invariom refinement The Hansen and Coppens (Hansen & Coppens, 1978) multipole parameters were transferred from the new def2TZVP Invariom database (see section 2.1 "Theoretical computations and new model compounds"in the main article) using the program InvariomTool (Hübschle *et al.*, 2007), including entries for the ligand(s), solvent(s) and counter ion atoms. The molecular charge was assigned using the default option three, which adjusts the monopole population according to the electronegativities of the elements. The model, including fixed aspherical scattering factors, was refined against F using XDLSM. Hydrogen positions were set to theoretical bond lengths of the respective model compound from the invariom database and

[2] All of those details are also true for refinements with SHELXL but did not need to be taken care of manually.

constrained to ride on their parent atoms.

Metal-complex specific theoretical electron density A single-point calculation for the metal complex with the coordinates found after the invariom refinement yielded a molecular wave function. The program GAUSSIAN09 (Frisch *et al.*, 2013) was employed for the DFT calculation with the functional M06 (Zhao & Truhlar, 2008) and basis set def2TZVP (Weigend & Ahlrichs, 2005). For the pairs of metal-complex structures investigated, the coordinates from the invariom refinement against the data set that yielded the best refinement result were used. For cases containing atoms on special positions, the molecule was completed prior to the single point calculation. The missing Cartesian coordinates were calculated with PLATON. The spin multiplicity, required for the single-point calculation, was initially deduced from ligand field theory. If high- and low-spin states were possible in principle, these were considered in different DFT calculations to confirm the favored multiplicity.

For the structures investigated the Self-Consistent Field (SCF) energies at the experimental geometries confirmed high-spin states for cobalt(II) and nickel(II) in the octahedral complexes as the favored electronic configurations (see Table 1). In the two instances of tetrahedral coordination (see Table 2) nickel(II) was lower energetically in its high-spin state for both cases. Cobalt(II) was only considered once for a tetrahedral complex, for which the high-spin state, originally deduced from crystal-field theory, was also confirmed by the quantum-mechanical computation. For the first three of four square-planar complexes, the low-spin state was favored for both metals (see Table 3), so that for the last one the DFT calculation was only performed for the low-spin state to generate the scattering factors for the 'whole molecule'.

For those cases treated first,[3] scattering factors were generated and compared for

[3] The chronological order differed from the numbering scheme, which was chosen only for better comprehensibility.

both spin states as well as taking the different metals into account. The comparisons, however, did not yield any valuable information, since, with exception of one case, the spin state variation affected only the incorrect metal. Therefore, later refinements were performed only for the energetically preferred spin states.

Table 1. *SCF energies in Hartree for the geometries refined with the invariom model for high- and low-spin cobalt(II) and nickel(II) in octahedral complexes. Lower energies are marked in bold.*

pair	Co(hs)	Co(ls)	Difference (ls-hs)
3	**-2687.4064**	-2687.3897	0.0167
4	**-2830.2233**	-2830.2019	0.0214
	Ni(hs)	Ni(ls)	
1	**-2724.3078**	-2724.2855	0.0223
3	**-2812.9664**	-2812.8447	0.1217
4	**-2955.7825**	-2955.7155	0.0670

Table 2. *SCF energies in Hartree for the geometries refined with the invariom model for high- and low-spin cobalt(II) and nickel(II) in tetrahedral complexes. Lower energies are marked in bold.*

pair	Co(hs)	Co(ls)	Difference (ls-hs)
8	**-7799.3570**	-7799.3111	0.0459
	Ni(hs)	Ni(ls)	
8	**-7924.9005**	-7924.8531	0.0473
10	**-3770.9427**	-3770.8689	0.0738

Table 3. *SCF energies in Hartree for the geometries refined with the invariom model for high- and low-spin cobalt(II) and nickel(II) square-planar complexes. Lower energies are marked in bold.*

pair	Co(hs)	Co(ls)	Difference (ls-hs)
5	-2378.4417	**-2378.4821**	-0.0404
	Ni(hs)	Ni(ls)	
5	-2504.0105	**-2504.0531**	-0.0426
6	-8662.2079	**-8662.2306**	-0.0227
7	-2928.3477	**-2928.3619**	-0.0142

Generation of 'simulated' structure factors The EDD of each molecule was placed at fractional coordinates at the approximate fractional coordinates $\frac{1}{4}$, $\frac{1}{4}$, $\frac{1}{4}$ in an artificial triclinic (seemingly cubic) unit cell with all lattice constants equal to 30 Å. To

avoid problems with flexible phases in non-centrosymmetric space groups in the multipole model (El Haouzi *et al.*, 1996), the space group was set to $P\bar{1}$. In contrast to space group $P1$, $P\bar{1}$ does not require calculation of the complete Ewald sphere. Since $P\bar{1}$ is centrosymmetric, Friedel pairs are identical and the wavelength dependent parts of the atomic scattering contributions were not included in the 'simulated' structure factors. We call them simulated, since these theoretical structure factors are subsequently used as observed structure factors in the least-squares refinement of multipole parameters. The structure factors were calculated from $h = k = -40$ and $l = 0$ to $h = k = l = 40$, giving a resolution of 1.15 in $\sin\theta/\lambda$ with the unit cell parameters given above. No thermal smearing or extinction was added, so that the subsequent refinement step concentrated on the asphericity of the EDD.

Refinement of the aspherical model against 'simulated' data Least-squares refinement of aspherical scattering factors against the 'simulated' theoretical data was carried out with the program XDLSM (from XD version 6.03). The choice of multipoles to be refined was based on local molecular site symmetry up to the next-nearest neighbors. The primary axis of the local coordinate system for the atoms engaged in a dative bond formation with the metal center were oriented towards the metal. Contraction parameters κ were refined for all atoms, while for atoms heavier than carbon κ' was also refined. Refinements were performed against F in as many cycles as were necessary to achieve convergence with a maximal shift/s.u. of 10^{-5}.

Refinement of the aspherical model for the 'whole molecule' The multipole parameters refined for the 'whole molecule' against the theoretical data were stored in a molecule-specific scattering-factor database by the developer version of the program InvariomTool. With this database file InvariomTool can transfer these parameters to the experimental structure models. All other settings for the refinement of the

'whole-molecule' scattering factors against the experimental data (last box in Figure 1 in the main article) were equal to those of the invariom refinements.

Geometry optimization In a few cases a geometry optimization was performed in addition to the single-point calculation in order to observe the changes that occurred upon geometry relaxation. DFT energies by themselves can not be compared between different metals for the complexes, as the number of electrons and thus the total SCF energy are different. The relative change upon geometry relaxation however, could indicate that for a particular metal the coordinates from the crystal structure are at, or close to, an energy minimum, whereas for the other metal a different atomic arrangement would be preferred. Hence, in some selected cases, the findings from the invariom-like approach are supported by geometry optimizations. For single-point as well as geometry optimization calculations the SCF convergence criterion was 10^{-8} a.u. and the integration grid pruned (99,950). DFT functional and basis set used were consistently M06/def2TZVP.

Table 4. *Unit-cell parameters for both structures of pair **3** and their estimated standard deviations.*

cell	*a*	*b*	*c*	beta
**a**	9.1863	13.8653	19.987	93.604
**b**	9.1896	13.8731	20.001	93.641
e.s.d.	0.0006	0.0009	0.001	0.001
Δ	0.0033	0.0078	0.014	0.037

Table 5. *Energy change upon geometry relaxation for pair **3**, in atomic units if not indicated otherwise. E(M06) is the energy from the calculation with the M06 density functional.*

	Ni(ls)	Ni(hs)	Co(hs)	Co(ls)
E(M06) starting geometry **a**	-2812.8400	-2812.9664	-2687.4064	-2687.3370
E(M06) starting geometry **b**	-2812.8984	-2812.9658	-2687.4055	-2687.3903
E(M06) optimized geometry		-2812.9664	-2687.4320	-2687.4161
quotient **a**		1.0000000	1.0000095	1.0000294
quotient **b**		1.0000002	1.0000098	1.0000095798
change [%]		0.0000%	0.0010%	0.0029%
change opt-sp		0.0000	-0.0256	-0.0791
change [kJ/mol]		0	-67	-208

1.2. Pair 2: Bis[2-(2-hydroxybenzoylhydrazono)propionato]nickel(II)/copper(II)

This octahedral complex **2** is similar to case **1**. The metals considered are copper(II) (Liu *et al.*, 2007) and nickel(II) (Wu *et al.*, 2007), as before. In addition to the complex, the unit cell contained three solvent water molecules, one of which forms a hydrogen bond to the O(4) of the ligand. Otherwise hydrogen bonds link the water molecules to one another and to those oxygen atoms of the complex that are not involved in metal coordination.

Table 6. *Structural formula and selected crystallographic and chemical information for pair 2.*

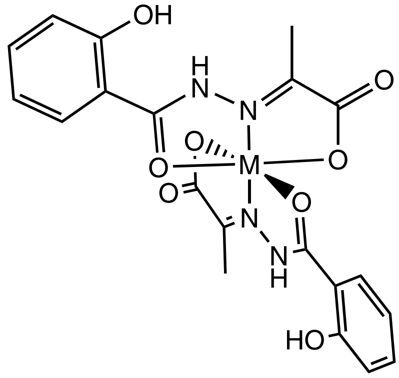

M = Ni / Cu

	**a**	**b**
Literature	(Wu *et al.*, 2007)	(Liu *et al.*, 2007)
IUCr code	dn2235	hy2076
CSD code	WINVOF	XILWUL
CCDC No.	669120	663160
Space group	$P\bar{1}$	
Peculiarity	3 water in a.u.	
Coord. geom.	distorted octahedral	
Metal ion	Ni^{2+}	Cu^{2+}
Electron config.	$[Ar]4s^0 3d^8$	$[Ar]4s^0 3d^9$
Spin multiplicity	3	2

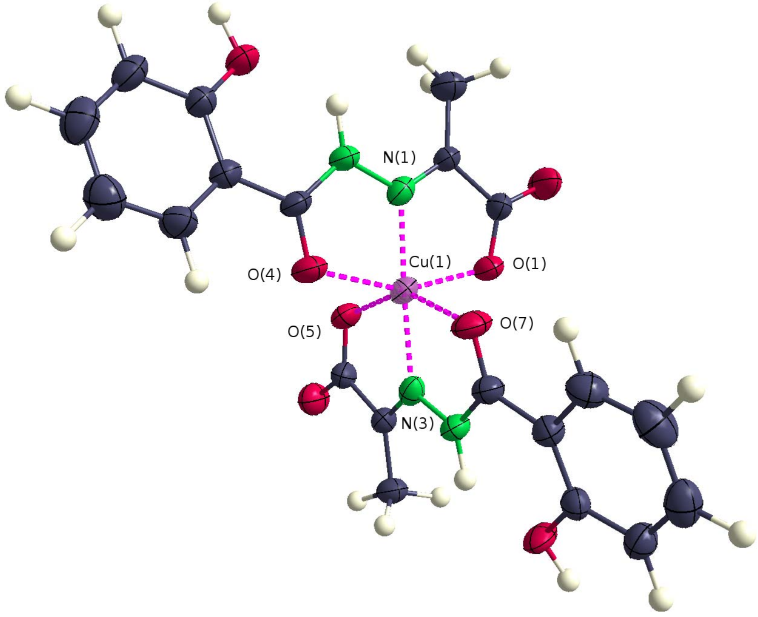

Fig. 1. Displacement ellipsoid plot at a probability of 50 % of the complex for pair **2** with the metal shown as Cu after refinement of the whole-molecule scattering factors against data set **b**. Water molecules are omitted for clarity.

Chemical reasoning A tetragonal distortion of the octahedron could in principle be caused by strain in the ligand, so JT splitting would not necessarily be the only explanation if the coordinating nitrogen atoms aligned to the principal tetragonal axis. However, an investigation of the bond distances around the central atom (see Table 1.2) reveals that the bonds to O(5) and O(7) are longer than those to the oxygen atoms O(1) and O(4), suggesting a JT distortion along the O(5)O(7) axis (see Figure 1).

Cu(1)—N(1)	1.929(3)
Cu(1)—N(3)	1.974(3)
Cu(1)—O(1)	2.042(3)
Cu(1)—O(4)	2.090(3)
Cu(1)—O(5)	2.208(3)
Cu(1)—O(7)	2.275(3)

A JT distortion is reasonable for the Cu complex with a d^9 electron configuration, but is less likely for the nickel ion (d^8). These bond length differences were clearly evident in the standard structure model produced by SHELXL.

Crystallographic aspects and refinement results The data for pair **2** are the same in both structures, differing only by a scale factor. Analysis using the deposited XRD data was sufficient to show that copper is the correct metal atom. Figure 2 shows the variation in the R(F) values with increasing aspherical modeling. The usual trend of improvement for the heavier metal can be seen. In contrast to most other cases, the heavier metal, Cu, gave the best fit in using the IAM model alone. The invariom and whole molecule refinements increased this differentiation significantly. Interestingly, inclusion of the multipole parameters for the metal atom decreased the R(F) for the nickel model also. This is however perfectly reasonable as the EDD for the system had not been modeled previously. The difference between the 'whole-molecule' scattering factors of 5.42 % and 5.01 % for **a** and **b**, respectively (Table 7), unambiguously proves that the metal is copper as this yields the best fit to the XRD data.

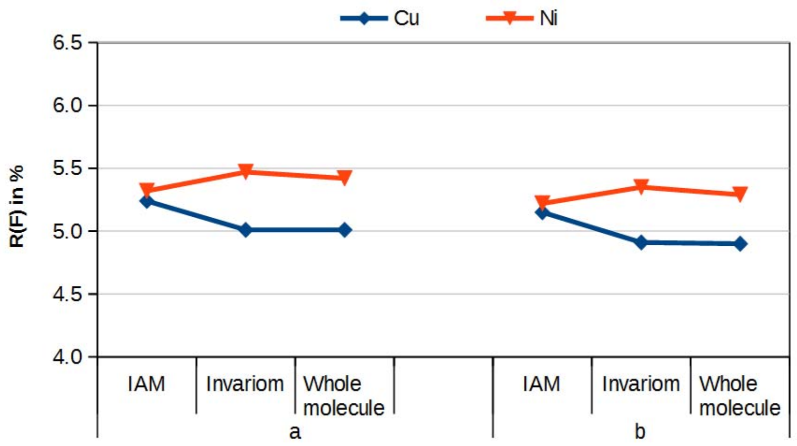

Fig. 2. Comparison of R(F) values for the refinements of the different metal atoms with different EDD models against the two data sets for pair **2**.

Table 7. *Selected computational and refinement results for pair 2.*

	Ni	Cu
DFT energy [au]	-3101.82	-3233.97
R(F) against theor. data	0.52	0.47
R(F) whole molecule a	5.42	**5.01**
R(F) whole molecule b	5.29	**4.90**

1.3. Pair 3: (5,5,7,12,12,14-Hexamethyl-1,4,8,11-tetraazacyclotetradecane-1,8-diacetato)-nickel(II)/cobalt(II)

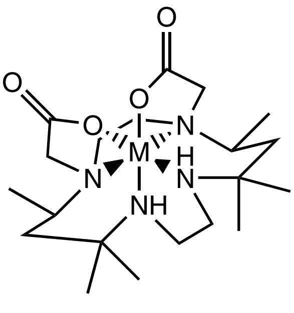

complex	3	
	**a**	**b**
Literature	(Wang *et al.*, 2005*b*)	(Wang *et al.*, 2005*a*)
IUCr code	ci6607	hb6232
CSD code	TAQSUA	CASHIO
CCDC No.	282277	282363
Space group	$P2_1/n$	
Peculiarity	4 water in a.u.	
Coord. geom.	octahedral	
Metal ion	Ni^{2+}	Co^{2+}
Electron config.	$[Ar]4s^03d^8$	$[Ar]4s^03d^7$
Spin multiplicity	3	2(ls) 4(hs)

Table 8. *Structural formula and selected crystallographic and chemical information for pair 3.*

Octahedral nickel(II) (Wang *et al.*, 2005*b*) or cobalt(II) (Wang *et al.*, 2005*a*) complexes with a chelating hexadentate ligand make up the pair of complexes considered here. The ligand provides four neutral nitrogen atoms and two negatively charged oxygen atoms that are part of acetate groups as the electron donors. The compound crystallized in the space group $P2_1/n$ and there are four non-coordinating water molecules in the asymmetric unit. In structure **b** one of these solvent molecules was modeled as partially occupied. The data sets are not completely equivalent and their unit cells also differ significantly (Table 4, Appendix A). In this case therefore both the identity of the metal and whether or not the metal is the same or different in each structure needs to be investigated.

Refinement results I In the IAM model, including the ionic scattering factors and including electron counts for the metal atoms, led to a lower R(F) for cobalt of 5.83 % than nickel 5.94 % for data set **a**. For data set **b** the absolute residuals are higher, but the ordering of the metals is similar. Upon introducing the invariom scattering factors the relationship of the metals changed for both data sets. With nickel as the metal, invariom modeling led to a significant improvement of R(F), whereas with cobalt the improvement was less for data set **a** while the fit was worse for data set **b** (Figure 3).

Spin state Before moving on to the 'whole-molecule' results, the input parameters for the DFT computation will be discussed briefly here. The energy comparisons for different spin states strongly confirmed the deduction from crystal field theory that nickel has a spin multiplicity of three in this octahedral coordination environment. When using geometry **a** the energy with low-spin nickel was -2812.84 or, for geometry **b**, -2812.90 Hartree. This needs to be compared to high spin nickel with -2912.97 Hartrees, giving certainly well over 180 kJ/mol in energy difference. Therefore only the ls spin state was evaluated further in Table 9. For cobalt the high-spin state is also energetically favored, at least in a gas phase calculation of the isolated molecule, but the EDD of the low-spin state was also projected onto the multipole model and compared to the experimental data, because the energy difference between the spin states was an order of magnitude smaller than that for nickel.

Refinement results II For data set **a**, which was published as a nickel complex, introduction of aspherical scattering factors for the whole molecule improved the density fitting still further. Although the improvement is similar for both metals, nickel clearly is a better model for the data (Figure 3).

For data set **b** the general data quality is worse. Different water models were tested, none of which improved the results significantly. Improvements for both metals were small for this data set but nickel again seemed to provide the best fit to the experimental EDD. Since the water molecules were less well identified in this structure, the solvates, rather than the complex might have a more significant influence on the quality of the fit. In this case using aspherical scattering factors to identify the metal atom may be less effective. A good structure model is clearly a prerequisite and this case demonstrates a limitation of the method.

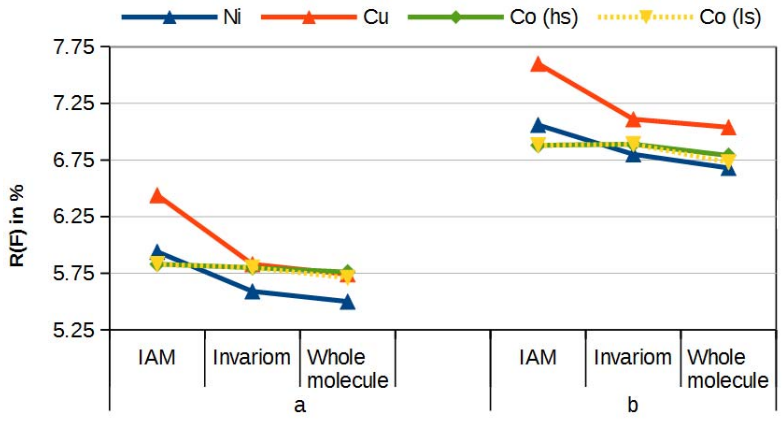

Fig. 3. Comparison of R(F) values for the refinements of the different metal atoms with different EDD models against the two data sets for pair **3**. Comparable values with Cu as the central metal atom are also shown.

Comparison to copper Since all of the pairs of structures investigated have shown that the heavier metal copper appears to be preferred on the basis of invariom treatment alone, we additionally included copper as the central metal atom in this particular complex. The results obtained for copper show that this preference for the heavier element does not occur in this case. The R(F) profiles comparing cobalt, nickel and copper show that while the invariom scattering factors improve for the copper structure, the nickel model was clearly the preferred metal of the three possibilities.

Table 9. *Selected computational and refinement results for pair 3. E(M06) is the energy from the calculation with the M06 density functional.*

	Ni(hs)	Co(hs)	Co(ls)	Cu
E(M06) crystal geometry [a.u.]	-2812.97	-2687.41	-2687.34	-2945.10
R(F) against theo. data [%]	0.48	0.49	0.51	0.48
R(F) whole molecule a [%]	**5.50**	5.76	5.71	5.74
R(F) whole molecule b [%]	**6.68**	6.79	6.73	7.04

Energetic considerations Since some DFT calculations had been performed during the procedure a theoretical backing for the decision on the correct metal in data set **b** was considered helpful. Therefore, the relative change upon relaxation of geometry was also taken into consideration. The nickel structure of the invariom model for **a**

was already at an energy minimum, while cobalt can be minimized by several kJ/mol (Table 5 in Appendix A). The crystal structure atomic coordinates are farthest away from those of the optimized low-spin cobalt complex and not at the minimum for cobalt high-spin state either. This was expected for data set **a**, where nickel had been determined to be the best fitting metal, while for **b** the situation was less clear. Especially, since the difference between low- and high-spin cobalt indicates a better fit for the low-spin state although it energetically appears as less stable in the gas phase. The difference between R1 of different spin states is almost as large as the difference between R1(all) of the Co(ls) model and the Ni model for data set **b**. Therefore, the support of nickel as the correct metal by the significantly larger change in energy upon geometry relaxation for cobalt in either spin state than for nickel was reassuring.

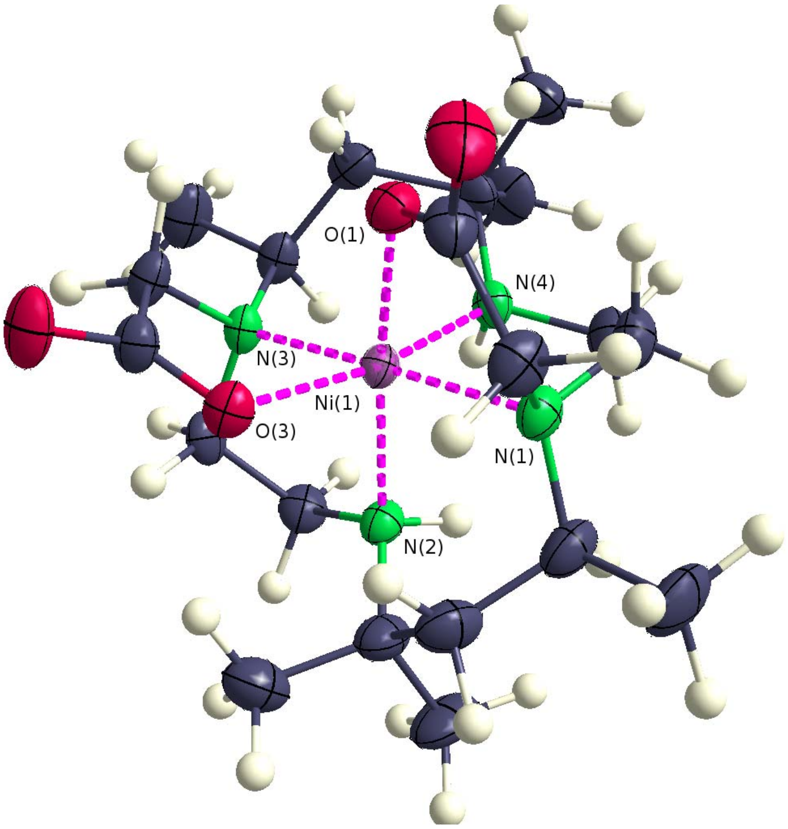

Fig. 4. Displacement ellipsoid plot at a probability of 50 % for the Ni comples of pair **3** after refinement of the whole-molecule scattering factors against data set **a**. Water molecules are omitted for clarity.

Evaluation of bond distances As Table 10 shows the largest deviation from the mean bond length to nickel was found for N(3) and N(1) that are *trans* to one another (Figure 4), with Ni-N1 longer and Ni-N3 shorter than the average. N(2) and N(4), the other two chemically equivalent nitrogen atoms, are both *trans* to oxygen atoms. However, none of the *trans*-oriented bond pairs show the considerable variation in length that would be expected to result from JT distortion. Furthermore, displacement ellipsoids of the ligand donor atoms displayed in Figure 4 show no elongation in the direction of the dative bonds. This further points to nickel being the correct metal ion rather than cobalt which, in it's low-spin state, would benefit from splitting of the e_g

orbitals, whereas in its high-spin state the splitting of t_{2g} orbitals would be slightly favored.

Comparison to the bond lengths from geometry optimizations shows a slightly greater shortening especially of the metal-N(3) bond for cobalt than for nickel. The two bonds to N(2) and N(4) also shortened in the geometry optimizations but to a similar extend for both metals.

Table 10. *Selected bond lengths for pair **3a** with nickel after refinement with whole-molecule scattering factors.*

atom pairs	crystal	geom opt Ni	geom opt Co ls
Ni(1)—N(1)	2.113(2)	2.11141	2.11011
Ni(1)—N(2)	2.102(2)	2.09189	2.09184
Ni(1)—N(3)	2.094(2)	2.08105	2.07935
Ni(1)—N(4)	2.103(2)	2.09449	2.09437
Ni(1)—O(1)	2.107(2)	2.10836	2.10779
Ni(1)—O(3)	2.113(2)	2.11409	2.11345

Conclusion In summary, chemical reasoning, bond length and energetic considerations and refinements with aspherical scattering factors confirm nickel as the correct metal in both structures.

1.4. Pair 5: [N,N′-Bis(3-methylsalicylidene)-1,3-propanediaminato]nickel(II)/cobalt(II)

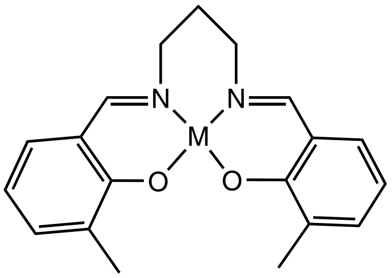

M = Ni / Co

Pair	**5**	
	**a**	**b**
Literature	(You, 2005*b*)	(Chen, 2006)
IUCr code	ci6619	sg6048
CSD code	TAQVEN	SAYZAU
CCDC No.	282297	296650
Space group	$A2_1am$	
Peculiarity	M and C10 on special position	
Coord. geom.	square planar	
Metal ion	Ni^{2+}	Co^{2+}
Electron config.	$[Ar]4s^0 3d^8$	$[Ar]4s^0 3d^7$
Spin multiplicity	1(ls) (3 hs)	2(ls) (4 hs)

Table 11. *Structural formula and selected crystallographic and chemical information for pair **5**.*

Structure **5a** (You, 2005*b*) was published in 2005 with nickel as the metal ion and structure **5b** (Chen, 2006) as a cobalt complex. The structures are isotypic. Both

square-planar complexes show mirror symmetry with a tetra-dentate ligand that coordinates *via* two anionic oxygen and two nitrogen atoms. The two data sets are discrete, but the fact the fact that they were discrete isotypic complexes with different central metal atoms was questionable, because the two unit cells are identical within the uncertainty limits.

Chemical reasoning Nickel(II) usually forms square-planar four coordinate complexes, while the equivalent geometry for cobalt(II) is either tetrahedral or octahedral. In a CSD search for complexes that at least contain this ligand and either nickel or cobalt as the central atom, the cobalt complexes that were found always had at least one more coordinative bond. Nickel on the other hand was shown to form several square-planar complexes with very similar ligands. Indeed an entry for the identical Ni complex with an additional methanol solvent molecule was found (TAQTUB). Therefore, nickel is more likely to be the correct central atom.

Spin state Theoretically the spin state of cobalt(II) in square-planar complexes would most likely be low spin. An explicit DFT calculation with the geometry obtained from the invariom refinement against data set **a** shows (Table 12) that the low-spin complex for cobalt is by 0.0404 Hartree (106kJ/mol) more stable in the gas phase. From the equivalent calculation for nickel, the low-spin configuration is also found to be of lower energy.

Table 12. *Selected computational and refinement results for pair* ***5****. E(M06) is the energy from the calculation with the M06 density functional.*

	Ni(ls)	Ni(hs)	Co(ls)	Co(hs)
E(M06) crystal geometry [a.u.]	**-2504.0531**	-2504.0105	**-2378.4821**	-2378.4417
R(F) against theo. data [%]	0.42	0.46	0.45	0.47
R(F) whole molecule a [%]	**3.34**	3.36	3.51	3.62
R(F) whole molecule b [%]	**3.39**	3.40	3.58	3.67

Crystallographic refinement From a crystallographic point of view the non-standard space group setting is noteworthy with two atoms on a special position of symmetry

m. Thus the z coordinates of these atoms were not refined and U_{23} and U_{13} were constrained to zero. Floating origin constraints for this space group also require fixing of x and y coordinates for one atom during refinement in XD. SHELXL and IAM refinements in XD gave a lower R-factor for cobalt than for nickel.

Refinement with scattering factors from the invariom database reversed the relation of the R(F) values between cobalt and nickel, now favoring nickel in both data sets (see Figure 5).

Refinement against the theoretical data showed no mirror symmetry for the oxygen donor atom. The multipoles at the metal were chosen according to the mirror symmetry at the metal atom. Refinement of otherwise equivalent multipole populations showed much better modeling with Ni as the central atom in comparison to Co. For both metals the low-spin state was better described by the multipole model than the high-spin data (Table 12).

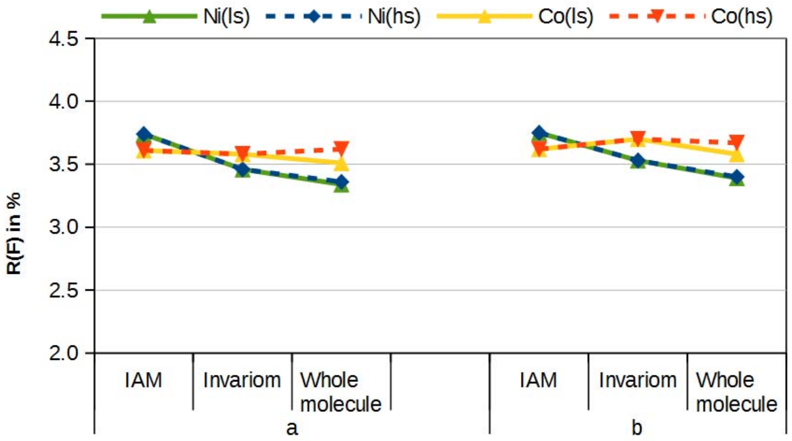

Fig. 5. Comparison of R(F) values for the refinements of the different metal atoms with different EDD models against the two data sets for pair **5**.

As shown in Figure 5 the 'whole-molecule' scattering factors refined against the experimental data of both structures significantly improved the R(F) values of the invariom refinement for nickel (hs and ls). For cobalt in the low-spin state the whole-

molecule result improved slightly with respect to the invariom result, too, but was worse than for nickel by approximately the same difference in R(F). High-spin cobalt yielded the worst result of all the 'whole-molecule' refinements. The multipole parameters for low-spin nickel yielded the best results but the gap between the spin-states is small. For cobalt spin considerations were no longer of interest, as both crystals clearly contain nickel.

1.5. Pair 6: Dibromidobis(2-methyl-5-phenyl-s-triazolo[3,4-b][1,3,4]thiadiazole-κN)nickel(II)/-copper(II)

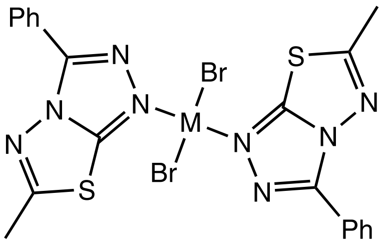

Pair	6	
	**a**	**b**
Literature	(Wang, 2007)	(Liu, 2007*b*)
IUCr code	bg2086	cf2132
CSD code	KIKBEM	XILXEW
CCDC No.	660111	664184
Space group	$P\bar{1}$	
Peculiarity	M on special position	
Coord. geom.	square planar	
Metal ion	Ni^{2+}	Cu^{2+}
Electron config.	$[Ar]4s^0 3d^8$	$[Ar]4s^0 3d^9$
Spin multiplicity	1(ls) (3 hs)	2

Table 13. *Structural formula and selected crystallographic and chemical information for pair* ***6****.*

This sixth pair of coordination compounds comprises a square planar nickel(II) or copper(II) complex with two bromide anions and two thiadiazole molecules as ligands. The thiadiazoles coordinate *via* a nitrogen atom. The data sets for the two structures **a** (Wang, 2007) and **b** (Liu, 2007*b*) are the same (**b** is 1.0309 times more intense than **a**) and the unit cell parameters are equal except for the uncertainty quoted for one angle. Thus, only one metal could be present and determination of the correct metal is the purpose of the investigation in this case.

Refinement I Interestingly, the atom with more electrons yielded better R(F) in the IAM refinements from the beginning. Therefore, the assignment of the central metal atom as copper(II) rather than nickel(II) was more likely from the beginning.

Chemical reasoning In this instance, chemical considerations alone could not differentiate between the metals. While bromide anions are weak-field ligands, nitrogen is a strong-field ligand. Accordingly copper(II) could adopt a square-planar geometry. A search of the CSD revealed that copper(II) complexes with two bromide ions and two nitrogen donating ligands can be found in both square-planar and tetrahedral coordination geometries.

Nickel(II) in square-planar geometry can be in a high or a low-spin state. The DFT energy of the low-spin state was less by 0.023 Hartree (60 kJ/mol) when the single-point energies for the refined geometries after invariom modeling are compared (see Table 14). Both spin states were included in the 'whole molecule' refinement procedures to investigate their influence on the aspherical models. The high-spin state could be described better by the multipole model, in contrast to the observation for pair **5**. There appears therefore to be no general rule concerning the multipole modeling of different spin states.

Table 14. *Selected computational and refinement results for pair **6**. E(M06) is the energy from the calculation with the M06 density functional.*

	Cu	Ni(hs)	Ni(ls)
E(M06) crystal geometry [a.u.]	-8794.271	-8662.208	**-8662.231**
R(F) against theo. data [%]	0.26	0.23	0.25
R(F) whole molecule a [%]	**5.12**	5.43	5.46
R(F) whole molecule b [%]	**4.87**	5.26	5.29

Refinement results Upon introduction of invariom scattering factors for the ligand the difference between the refinement results of the two metals stayed constant (Figure 6). Aspherical scattering factors for the whole molecules considerably improved the fits to both data sets for both metals, but the copper complex clearly provided the superior model for the data.

That the high-spin state of nickel marginally yielded better results than in the low-spin state can be explained by the population of similar orbitals for eight electrons,

if two are unpaired. In the low-spin case the highest occupied molecular orbital is different for nickel to that for copper, which makes the fit worse. But compared to the overall value of R(F), the gap due to the spin state is insignificant. The lower R(F) values for the second data set originated in the smaller number of reflections therein.

Data set **b** has fewer reflections, whereas set **a** includes reflections to a resolution of 0.77 Å. The two models including multipoles clearly led to the conclusion that copper is the correct metal in this case.

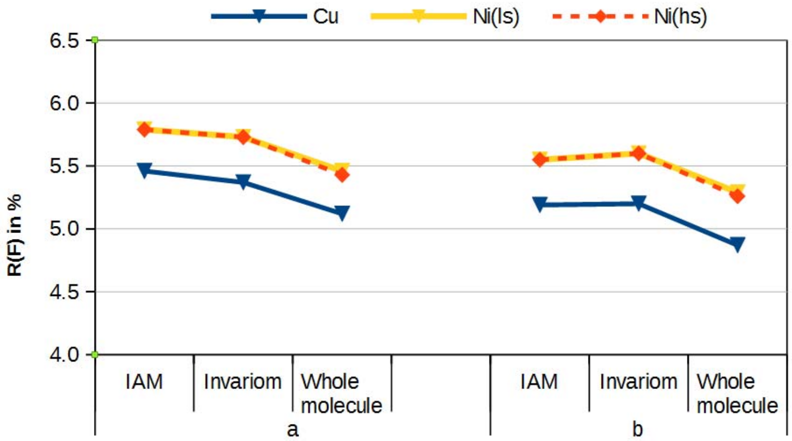

Fig. 6. Comparison of R(F) values for the refinements of the different metal atoms with different EDD models against the two data sets for pair **6**.

1.6. Pair 7: Bis[6-(cyclopentyliminomethyl)-2-methoxyphenolato]nickel(II)/copper(II)

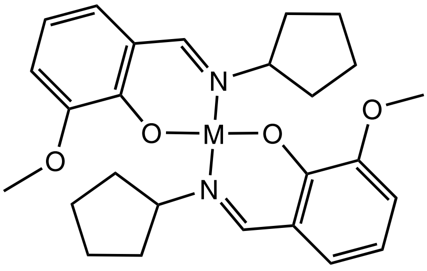

M = Ni / Cu

Pair	**7**	
	**a**	**b**
Literature	(Zhao, 2007)	(Hou, 2007)
IUCr code	cf2085	gk2077
CSD code	SEZFIN	YIDRUZ
CCDC No.	640293	650575
Space group	*Pbca*	
Peculiarity	M on special position ($\bar{1}$)	
coord. geom.	square planar	
Metal ion	Ni^{2+}	Cu^{2+}
Electron config.	[Ar]$4s^0 3d^8$	[Ar]$4s^0 3d^9$
Spin multiplicity	1(ls) (3 hs)	2

Table 15. *Structural formula and selected crystallographic and chemical information of pair* ***7****.*

Complex pair **7** is similar to pair **6**. The complexes are square-planar, crystallize with the metals on inversion centers and were published as a nickel (Zhao, 2007) and a copper (Hou, 2007) compound. The reflection data are however discrete for **a** and **b**. This allows the possibility that both structures contain different metals. As Table 16 shows, the unit cell parameters *b* and *c* differ somewhat. The unit cell volume of **a** is greater than that of **b**, but the data collection temperature for **a** was reported to be 5 K higher than for **b**. This temperature difference could explain the variation in cell constants and volumes. The crystal quality of the data for **a** appeared higher, with more reflections and lower cell e.s.d.s. Figures of merit (except for the Goof) reported were superior in comparison to data set **b**.

Table 16. *Unit-cell parameters for the two datasets of pair* ***7***.

Cell	*a*	*b*	*c*	T [K]
**a**	12.0620(10)	10.8110(10)	17.887(2)	298(2)
**b**	12.060(2)	10.8025(18)	17.863(3)	293(2)

Refinement results Comparing the IAM models for both metals, nickel(II) yielded a lower R(*F*) than copper for both data sets (see Figure 7). However, on inclusion of the invariom scattering factors, the model with the heavier element, copper, improved considerably in both cases. The improvement was better for data set **a** but was also observed for the other data set. Including aspherical scattering factors for the metals provided some technical difficulties and in the end no further improvement was observed for either metal, but both data sets were modeled much better with copper as the central metal atom. For data set **a** the difference for nickel in either spin state increased, and although the gap between the models with the different metals was smaller for **b**, it was still significant. Energetically (Table 17) the low-spin state of nickel would be favored in this coordination geometry.

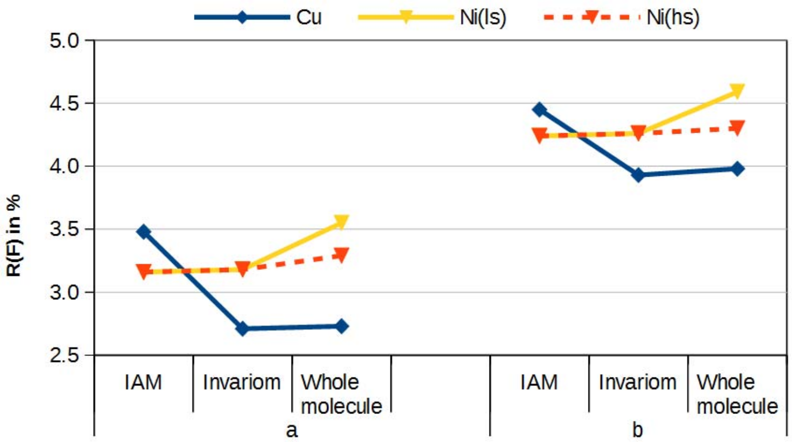

Fig. 7. Comparison of R(F) values for the refinements of the different metal atoms with different EDD models against the two data sets for pair **7**.

Overall, it was clear that copper was the correct metal atom in both structures. Although the data sets differed by more than a scale factor, aspherical scattering factors showed clearly that copper fitted better to both sets of diffraction data than nickel.

Table 17. *Selected computational and refinement results for pair* **7**. *E(M06) is the energy from the calculation with the M06 density functional.*

	Cu	Ni(hs)	Ni(ls)
E(M06) crystal geometry [a.u.]	-3060.527	-2928.348	-2928.362
R(F) against theo. data [%]	0.49	0.46	0.52
R(F) whole molecule a [%]	**2.73**	3.29	3.55
R(F) whole molecule b [%]	**3.98**	4.30	4.59

1.7. Pair 10: Dichlorobis[2-(o-tolyliminomethyl)phenolato]nickel(II)/copper(II)

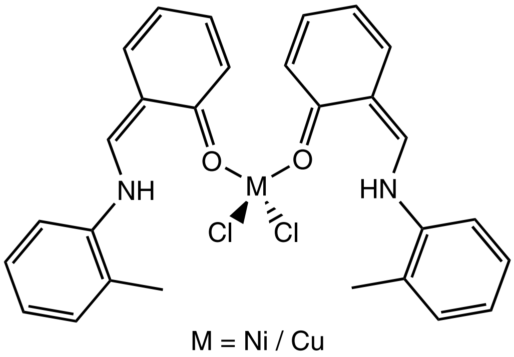

Table 18. *Structural formula and selected crystallographic and chemical information of pair* ***10****.*

	a	b
Literature	(Yang, 2005*b*)	(Liu & Zeng, 2006)
IUCr code	cf6444	is2046
CSD code	TAQREJ	ADIFID
CCDC No.	282269	613739
Space group	$C2/c$	
Peculiarity	M on special position (2)	
Coord. geom.	tetrahedral	
Metal ion	Cu^{2+}	Ni^{2+}
Electron config.	$[Ar]4s^0 3d^9$	$[Ar]4s^0 3d^8$
Spin multiplicity	2	3

Pair **10** is the second tetrahedral complex investigated and the metal is coordinated by two chloride anions and two oxygen atoms, which are part of an almost completely planar phenolate ligand. The complex with either nickel(II) (Liu & Zeng, 2006) or copper(II) (Yang, 2005*b*) crystallized with the metal atom on a twofold rotation axis. The reflection data are the same, except for a slightly lower resolution in set **b**; so in reality only one metal can be correct.[4]

Chemical reasoning Past results and chemical knowledge suggest that nickel(II) is more likely to be found in a tetrahedral coordination geometry than is copper(II).

[4] Something else that came to our attention when studying the compound was the placement of a double bond at the nitrogen in the structural formula. As it is presented in the literature (Yang, 2005*b*; Liu & Zeng, 2006) there should at least be a positive charge at the -NH=. A better representation of the structure is the formula depicted in Table 18, since although the bond drawn as a double bond has a length of 1.42566 Å, which is shorter than a common aromatic bond, the following bond to the nitrogen atom is even closer to a common carbon-nitrogen double bond [1.29 Å (Müller *et al.*, 2006)) with a length of 1.29 Å. Additionally, the carbon-oxygen bond is with 1.297 Å definitely closer to a double bond (1.21 Å (Müller *et al.*, 2006)) than a single bond (1.43 Å (Müller *et al.*, 2006)].

There have been cases of close-to-tetrahedral copper(II) complexes, (Kitajima *et al.*, 1990; Sacconi & Ciampolini, 1964) however, rendering a tetrahedron a *ćommonly observed geometry"* (Müller *et al.*, 2006) for copper. Multi-dentate ligands or counterions as in $Cs_2[CuCl_4]$ (Hollemann & Wiberg, 2007) can promote at least pseudo-tetrahedral arrangements, hence, copper is chemically possible as the metal in one of these complexes.

Bond angles for the 'whole-molecule' structure with nickel refined against dataset **a** are shown in Table 19. The bond angles indicate a larger deviation from a square-planar coordination geometry than from a tetrahedral one, but they neither belong to a perfect tetrahedron.

Table 19. *Selected bond angles from the final structure of pair* ***10***.

Cl(1)–Ni(1)—Cl(1)_1	110.22(9)
Cl(1)–Ni(1)—O(1)	106.25(10)
Cl(1)–Ni(1)—O(1)_1	115.76(11)
O(1)–Ni(1)—O(1)_1	102.7(2)

Refinement results For this structure the refinement results were not particularly good. With an R(F) higher than 6.5% for the higher resolution data set, these models had the worst fit to the XRD data of all cases investigated. Only the fit for **3b** was worse, but that was due to the presence of several disordered solvent water molecules. There are no such obvious explanations in this case, probably suggesting poor crystal quality. The highest difference density peaks were located around the metal atom and since the residual density map was featureless except in the region around the metal center, there was no disorder. The data are simply very noisy.

Nonetheless, an invariom refinement led to a greater improvement in the fit for the copper complex than for the nickel. The inclusion of aspherical scattering factors for the coordination center had quite different effects for set **a** and **b**, although they do not differ in the low order data. This raises the question of how accurately the inner data were determined. Nevertheless the fit to both sets of data was clearly the best

for copper(II) confirming that both structures are likely to be of copper complexes.

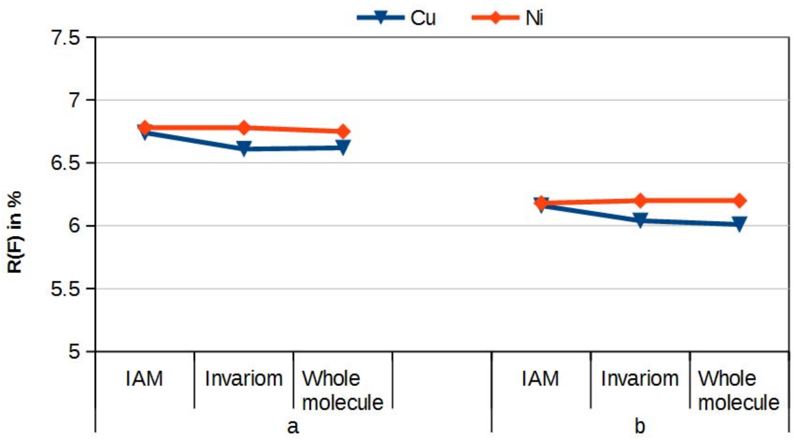

Fig. 8. Comparison of R(F) values for the refinements of the different metal atoms with different EDD models against the two data sets for pair **10**.

Results from theoretical investigation Since the crystallographic results did not agree well with the known coordination behavior of such metals and the data quality is low, the relative energies of both complexes were determined for atomic coordinates from the invariom and geometry optimized refinements. The energy change upon geometry relaxation was identical for high-spin nickel and copper, so that in this case only a slight preference of copper could be derived from the experimental data.

1.8. *Pair 11: Azido[1-(isobutylaminomethyliminomethyl)-2-naphtholato]nickel(II)/copper(II)*

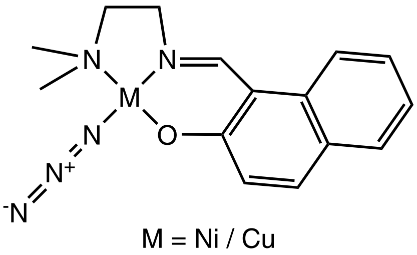

Pair	**11**	
	**a**	**b**
Literature	(Sun *et al.*, 2005*a*)	(Zhu *et al.*, 2006)
IUCr code	su6164	sj2024
CSD code	FEYXEN	TEJMAX
CCDC No.	263585	608486
Space group	*Pbca*	
Peculiarity	slight disorder of *en* ligand	
Coord. geom.	square planar	
Metal ion	Ni^{2+}	Cu^{2+}
Electron config.	$[Ar]4s^0 3d^8$	$[Ar]4s^0 3d^9$
Spin multiplicity	3	2

Table 20. *Structural formula and selected crystallographic and chemical information of pair **11**.*

For the azido[1-(isobutylaminomethyliminomethyl)-2-naphtholato] complex shown in Table 20 two structures, one with nickel(II) (Sun *et al.*, 2005*a*) and one with copper(II) (Zhu *et al.*, 2006), appear in the CSD. Both are square-planar complexes with a slightly disordered ethylenediamine ligand. The data sets are unique, but the unit cells are very similar (Table 21). A comparison of bond lengths and angles did not reveal any significant differences between the two supposedly different models refined against the different data sets either. Therefore, it is very likely that only one metal is correct. Copper(II) is more likely to adopt a square-planar coordination geometry and in this case the coordination geometry was not imposed by the ligand, since the azide could readily move out of the plane.

Table 21. *Unit-cell parameters in Å for pair **11** **a** and **b**, both measured at 298(2) K.*

	a	*b*	*c*
**a**	7.5760(10)	13.300(2)	30.306(2)
**b**	7.5680(10)	13.3060(10)	30.280(2)
Δ [Å]	0.008	0.006	0.026
Δ [%]	0.1	0.04	0.8

Refinement results This pair of structures was problematic due to the disorder in the ethylene group of the organic ligand. The second disorder component had an occupancy of less than 5%, but this was sufficient to hinder the correct element assignment from refinement results. The disorder could only be modeled by several

strong restraints in SHELXL, which could not be treated properly in XD (Volkov *et al.*, 2006). Since the disorder accounted for less than one electron, aspherical refinements without description of the disorder were attempted with XD.

However, hardly any improvement could be seen upon invariom modeling of the ligand environment as illustrated in Figure 9. Although the model containing copper, which was the poorer IAM model, improved more than the one with nickel, the latter remained superior in fitting the XRD data. The same trend was observed for the aspherical modeling around the metal center. Since this part of the model is farther away from the disorder, the improvement was greater than for the invariom model. Again, the copper model improved more than the nickel model, but the influence was not enough to show an obvious preference between the two metals.

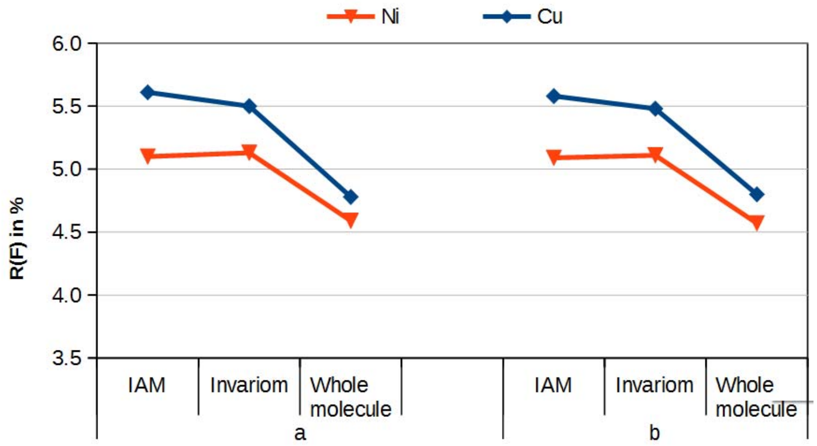

Fig. 9. Comparison of R(F) values for the refinement of the different metal atoms with different EDD models against the two data sets for pair **11**.

Disorder is clearly going to be a problem when an aspherical model is used to try to distinguish between two metals. Hence, this case provides an example of a limitation of this method.

Overall, the two data sets behaved very similar during the different refinements, supporting the hypothesis that only one metal is correct. The trends of the refinements

suggest copper as the better model if the disorder would not damp improvement. Unfortunately, the evidence based upon the diffraction data are not strong enough to prove that one or other structure is unambiguously correct.

References

Chen, Y. (2006). *Acta Cryst. E* **62**, m144–m145.

El Haouzi, A., Hansen, N. K., Le Hénaff, C. & Protas, J. (1996). *Acta Cryst. A* **52**, 291–301.

Frisch, M. J., Trucks, G. W., Schlegel, H. B., Scuseria, G. E., Robb, M. A., Cheeseman, J. R., Scalmani, G., Barone, V., Mennucci, B., Petersson, G. A., Nakatsuji, H., Caricato, M., Li, X., Hratchian, H. P., Izmaylov, A. F., Bloino, J., Zheng, G., Sonnenberg, J. L., Hada, M., Ehara, M., Toyota, K., Fukuda, R., Hasegawa, J., Ishida, M., Nakajima, T., Honda, Y., Kitao, O., Nakai, H., Vreven, T., Montgomery, J. A., Peralta, J. E., Ogliaro, F., Bearpark, M., Heyd, J. J., Brothers, E., Kudin, K. N., Staroverov, V. N., Keith, T., Kobayashi, R., Normand, J., Raghavachari, K., Rendell, A., Burant, J. C., Iyengar, S. S., Tomasi, J., Cossi, M., Rega, N., Millam, J. M., Klene, M., Knox, J. E., Cross, J. B., Adamo, V. B. C., Jaramillo, J., Gomperts, R., Stratmann, R. E., Yazyev, O., Austin, A. J., Cammi, R., Pomelli, C., Ochterski, J. W., Martin, R. L., Morokuma, K., Zakrzewski, V. G., Voth, G. A., Salvador, P., Dannenberg, J. J., Dapprich, S., Daniels, A. D., Farkas, O., Foresman, J. B., Ortiz, J. V., Cioslowski, J. & Fox, D. J. (2013). *Gaussian 09, Revision D.01.* Tech. rep. Gaussian, Inc., Pittsburgh PA.

Hansen, N. K. & Coppens, P. (1978). *Acta Cryst. A* **34**, 909–921.

Hollemann, A. F. & Wiberg, N. (2007). *Lehrbuch der Anorganischen Chemie.* Berlin: Walter de Gruyter, 102nd ed.

Hou, H.-Y. (2007). *Acta Cryst. E* **63**, m1766.

Hübschle, C. B., Luger, P. & Dittrich, B. (2007). *J. Appl. Cryst.* **40**, 623–627.

Kitajima, N., Fujisawa, K. & Moro-oka, Y. (1990). *J. Am. Chem. Soc.* **112**, 3210–3212.

Liu, F., Wu, W.-T., Zhang, W.-P., Chen, F.-Y. & He, S.-Y. (2007). *Acta Cryst. E* **63**, m2450–m2451.

Liu, H.-Q. (2007*b*). *Acta Cryst. E* **63**, m2466.

Liu, X.-H. & Zeng, J.-H. (2006). *Acta Cryst. E* **62**, m1501–m1503.

Müller, P., Herbst-Irmer, R., Spek, A., Schneider, T. & Sawaya, M. (2006). *Crystal Structure Refinement: A Crystallographer's Guide to SHELXL.* New York: Oxford University Press, 1st ed.

Prince, E. (ed.) (2004). *International Tables for X-ray Crystallography Volume C: Mathematical, Physical and Chemical Tables*, vol. C. Dordrecht: Kluwer Academic Publishers, 3rd ed.

Sacconi, L. & Ciampolini, M. (1964). *J. Chem. Soc. (Resumed)*, pp. 276–280.

Sheldrick, G. M. (2015*a*). *Acta Cryst. C* **71**, 3–8.

Spek, A. L. (2009). *Acta Cryst. D* **65**, 148–155.

Sun, Y.-X., Gao, G.-Z., Zhang, R. & Pei, H.-X. (2005*a*). *Acta Cryst. E* **61**, m397–m398.

Volkov, A., Macchi, P., Farrugia, L. J., Gatti, C., Mallinson, P., Richter, T. & Koritsánszky, T., (2006). *XD2006 – A Computer Program Package for Multipole Refinement, Topological Analysis of Charge Densities and Evaluation of Intermolecular Energies from Experimental or Theoretical Structure Factors.*

Wang, L.-G. (2007). *Acta Cryst. E* **63**, m2345.

Wang, S.-F., Ruan, B.-F., Li, H.-Q., Zhu, H.-L. & Ng, S. W. (2005*a*). *Acta Cryst. E* **61**, m1484–m1485.

Wang, S.-F., Xue, J.-Y., Shi, L., Zhu, H.-L. & Ng, S. W. (2005*b*). *Acta Cryst. E* **61**, m1481–m1483.

Weigend, F. & Ahlrichs, R. (2005). *Phys. Chem. Chem. Phys.* **7**, 3297–3305.

Wu, W.-P., Zeng, F.-C. & Wu, Y. (2007). *Acta Cryst. E* **63**, m2664.

Yang, Y.-M. (2005*b*). *Acta Cryst. E* **61**, m1616–m1617.
You, Z.-L. (2005*b*). *Acta Cryst. E* **61**, m1637–m1638.
Zhao, X.-F. (2007). *Acta Cryst. E* **63**, m704–m705.
Zhao, Y. & Truhlar, D. G. (2008). *Theor. Chem. Acc.* **120**, 215–241.
Zhu, Q.-Y., Wei, Y.-J. & Wang, F.-W. (2006). *Acta Cryst. E* **62**, m983–m985.
